# Long-Term Mortality and Morbidity After Pediatric Sepsis

**DOI:** 10.1093/ofid/ofag660

**Published:** 2026-10-07

**Authors:** Erik Edholm, Adam Linder, Malin Inghammar, Anton Nilsson, Sara Jesperson, Lisa Mellhammar

**Affiliations:** Division of Infection Medicine, Department of Clinical Sciences Lund, Lund University, Lund, Sweden; Department of Pediatrics, Central Hospital of Kristianstad, Kristianstad, Sweden; Division of Infection Medicine, Department of Clinical Sciences Lund, Lund University, Lund, Sweden; Department of Infectious Diseases, Skåne University Hospital, Lund, Sweden; Division of Infection Medicine, Department of Clinical Sciences Lund, Lund University, Lund, Sweden; Department of Infection Prevention and Control, Skåne University Hospital, Lund, Sweden; Epidemiology, Population Studies and Infrastructures EPI@LUND, Department of Laboratory Medicine, Lund University, Lund, Sweden; Department of Translational Medicine, Lund University, Malmö, Sweden; Clinical Studies Sweden–Forum South, Skåne University Hospital, Lund, Sweden; Division of Infection Medicine, Department of Clinical Sciences Lund, Lund University, Lund, Sweden; Department of Infectious Diseases, Skåne University Hospital, Lund, Sweden

**Keywords:** epidemiology, long-term effects, mortality, pediatric, sepsis

## Abstract

**Background:**

Adults experience sustained disease burden in the years following sepsis, but data regarding long-term prognosis after pediatric sepsis are still limited. The objective of this study was to determine long-term outcomes after sepsis in children, in terms of mortality, morbidity, and socioeconomic factors.

**Methods:**

This cohort study was based on a nationwide historical cohort of 55 906 pediatric sepsis hospitalizations, as compared with a control cohort of 93 274 pediatric nonsepsis hospitalizations with organ dysfunction, with linked register data on patient characteristics. Exposure was defined as sepsis hospitalization before 18 years of age. Cox regression was used to estimate hazard ratios for long-term mortality, with adjustments for age and comorbidities. Logistic regression was used to compare the prevalence of diseases in the groups during follow-up. Socioeconomic outcome in terms of education level, unemployment, and disability pension was compared with logistic regression at 20, 25, and 30 years of age. The study was set in Sweden, 1 January 1987 to 31 December 2022.

**Results:**

During the first 5 years of follow-up, the sepsis cohort had higher mortality, with a hazard ratio of 1.17 (95% CI, 1.11–1.22), adjusted for age and comorbidities. Subsequent sepsis, infectious disease, and respiratory disease during the follow-up were more common in the sepsis cohort than in the control cohort.

**Conclusions:**

The findings of this study call for increased attention of health care to survivors of pediatric sepsis and enhanced follow-up.

## Teaser

In this nationwide cohort study in Sweden, we found higher mortality after pediatric sepsis in the first 5 years after discharge, as compared with a control cohort of children hospitalized with organ dysfunction, indicating sustained disease burden a long time after discharge.

Sepsis is a serious disease, caused by an infection and a subsequent dysregulated immune response, which causes tissue damage and ultimately may lead to organ dysfunction and death [1, 2]. Mortality of sepsis in children ranges from 6% to 25%, although reported numbers differ depending on location and study methods [3–6]. Pediatric sepsis is a major health issue, globally affecting an estimated 4.2 million children yearly [4], resulting in a large number of young sepsis survivors. Studies on adults have shown that apart from acute complications or sequelae evident at an early stage or at discharge, sepsis survivors are subject to a range of complications that emerge a long time after the sepsis [7, 8]. The mechanisms and causal pathways for these phenomena are likely multifactorial and not yet fully understood. Emerging evidence on pediatric patients points to increased disease burden and readmissions in the months following discharge after sepsis [9, 10]. Follow-up from pediatric intensive care units has shown that almost a fifth of all children with sepsis had acquired a new disability after 3 months [3]. Moreover, a substantial proportion of pediatric sepsis survivors develop new or progressive medical conditions within 6 months [11]. However, as stated in a recent review article, existing data are limited beyond 12 months, stressing the need for further research [12]. Thus, with mounting evidence of long-term consequences after adult sepsis, there is a need to investigate the pediatric population.

The objective of the study was to investigate long-term mortality, morbidity, and socioeconomic status after sepsis in childhood.

## METHODS

We conducted a nationwide retrospective cohort study using health care data from the National Patient Register and the National Cause of Death Register, kept by the National Board of Health and Welfare in Sweden. Data on educational status, unemployment, and disability pension were obtained from the Swedish Longitudinal Integrated Database for Health Insurance and Labour Market Studies, kept by Statistics Sweden, and linked to health care data. National data were retrieved from 1 January 1987 to 31 December 2022 and included hospitalizations (length of stay, age at discharge, *International Classification of Diseases* [*ICD*] codes at discharge, time of discharge), episodes of specialized outpatient care (*ICD* codes, age, time of visit), and mortality (time of death, cause of death). The detail level on acquired data on patient age was years. No outpatient data were available before 2001, when specialized outpatient care data became available. Separate hospitalizations were combined if a patient had a new hospitalization within 1 day of discharge. If patients met inclusion criteria for 1 or both cohorts at multiple occasions, they were assigned the cohort to which they first met inclusion criteria. The study was granted permission by the Swedish Ethics Review Appeals Board (No. Ö 55-2021/3.1). The report was conducted according to the STROBE reporting guidelines (Strengthening the Reporting of Observational Studies in Epidemiology) [13].

### Sepsis Cohort Identification

The sepsis cohort was selected through explicit and implicit sepsis criteria. Patients were included if they were <18 years of age and had a hospitalization that (1) had an explicit *ICD* code for sepsis or (2) met implicit sepsis criteria—specifically, a concurrent *ICD* code for infection and organ dysfunction in the discharge letter [14] (Supplementary Table 1). Index dates were days of discharge. This combined method was chosen to enhance sensitivity for sepsis [15–17]. No further exclusion criteria were applied on the patients meeting the stated inclusion criteria. Selection was made with discharge *ICD* codes from hospitalizations per the Swedish modifications of the *International Classification of Diseases, Ninth Revision* (*ICD-9*) [18] and the *International Statistical Classification of Diseases and Related Health Problems, Tenth Revision* (*ICD-10-SE*) [19]. We used the selection of *ICD* codes for explicit and implicit sepsis previously compiled by Fleischmann-Struzek et al [16], which were adapted to *ICD-10-SE* and translated [20, 21] to the Swedish *ICD-9* (Supplementary Table 1). The start of the study period was chosen as 1987 because the National Patient Register reached national coverage in Sweden in this year [18].

### Control Cohort Selection

The control cohort without sepsis was selected from criteria of having a history of hospitalization before 18 years of age with an *ICD* code for organ dysfunction and concurrent absence of an *ICD* code for infection in the discharge letter (Supplementary Table 1). Index dates were days of discharge. The control cohort was chosen to compensate for the potential confounding effects of hospital care and for the groups to be as similar as possible apart from sepsis and thus reduce the impact of potential unknown confounders.

### Outcome Variables

The primary outcome was all-cause mortality. Secondary outcomes included new diseases, grouped by chapters in the *ICD* (Supplementary Table 2); cause of death, grouped by chapters in the *ICD*; and socioeconomic factors, including educational status, days of unemployment, and disability pension. *ICD-10-SE* codes were translated to the *ICD-9* [20, 21]. *ICD* codes for cause of death were collected and treated equally regardless of whether they were noted as the primary or underlying cause of death. The definition of sepsis-related cause of death was either an explicit sepsis *ICD* code or concurrent *ICD* codes for infection and organ dysfunction (Supplementary Table 1) in the death certificate. Education status was defined as the highest level of achieved education (Supplementary Table 3).

### Strategy for Adjustments

The groups were adjusted for age and comorbidities through *ICD* codes from previous hospitalizations and outpatient care. We used the Charlson Comorbidity Index (Supplementary Table 4), which has been evaluated in children [22], in a Swedish translation [23].

### Statistical Analysis

Statistical analysis was performed in R studio version 4.3.3. Baseline characteristics are presented as median and IQR for numeric variables and as absolute and relative frequency for categorical variables. Mortality is visualized with a Kaplan-Meier graph with separate survival curves for the sepsis and control cohorts. A Cox proportional hazards model, adjusted for comorbidities and age, was used to estimate the hazard ratio (HR) for sepsis hospitalizations as compared with controls, with time from index hospitalization as the time scale. The crude 30-day, 1-year, and 5-year mortalities were compiled for each group.

Sensitivity analyses for mortality were performed (1) on patients without neoplastic disease, (2) on 2 separate groups of patients diagnosed by years 1987–1996 (*ICD-9*) and 1997–2022 (*ICD-10-SE*), and (3) on the group with outpatient care data available, as well as by age categories: 0 to <1 year, 1 to <7, 7 to <13, 13 to <18, and 1 to <18. A sensitivity analysis regarding morbidity was performed on 0 to <1 and 1 to <18 years of age. Additionally, a competing risk analysis was performed to factor in death as a competing event to morbidity, based on a Fine-Gray subdistribution hazard model (Supplementary Table 5).

New diagnoses presenting after the index hospitalization were summarized as count and percentage, excluding preexisting diagnoses, and presented as an overall value based on time of diagnosis. Logistic regression was used to compare the prevalence of new diagnoses between the groups in different time windows for a prespecified selection of disease categories grouped by *ICD* chapters.

Education level, days of unemployment, and occurrence of disability pension were compared with logistic regression at 20, 25, and 30 years of age.

## RESULTS

A total of 149 180 hospitalizations were included in the study. Among them, 55 906 were identified as patients with sepsis, leaving 93 274 included in the control cohort (Figure 1). The sepsis cohort had a median age of 0 years (IQR, 0–1; Table 1), and the median age of the control cohort was also 0 years (IQR, 0–0). The sex distribution was similar, with 32 509 (58%) male patients in the sepsis cohort and 53 569 (57%) in the control cohort. At baseline, the number of patients with existing neoplastic disease was 3039 (5.4%) in the sepsis cohort and 624 (0.7%) in the control cohort. Missing data were <0.1% on any baseline variables at inclusion, and individuals with missing data were excluded from further analysis.

**Figure 1.**
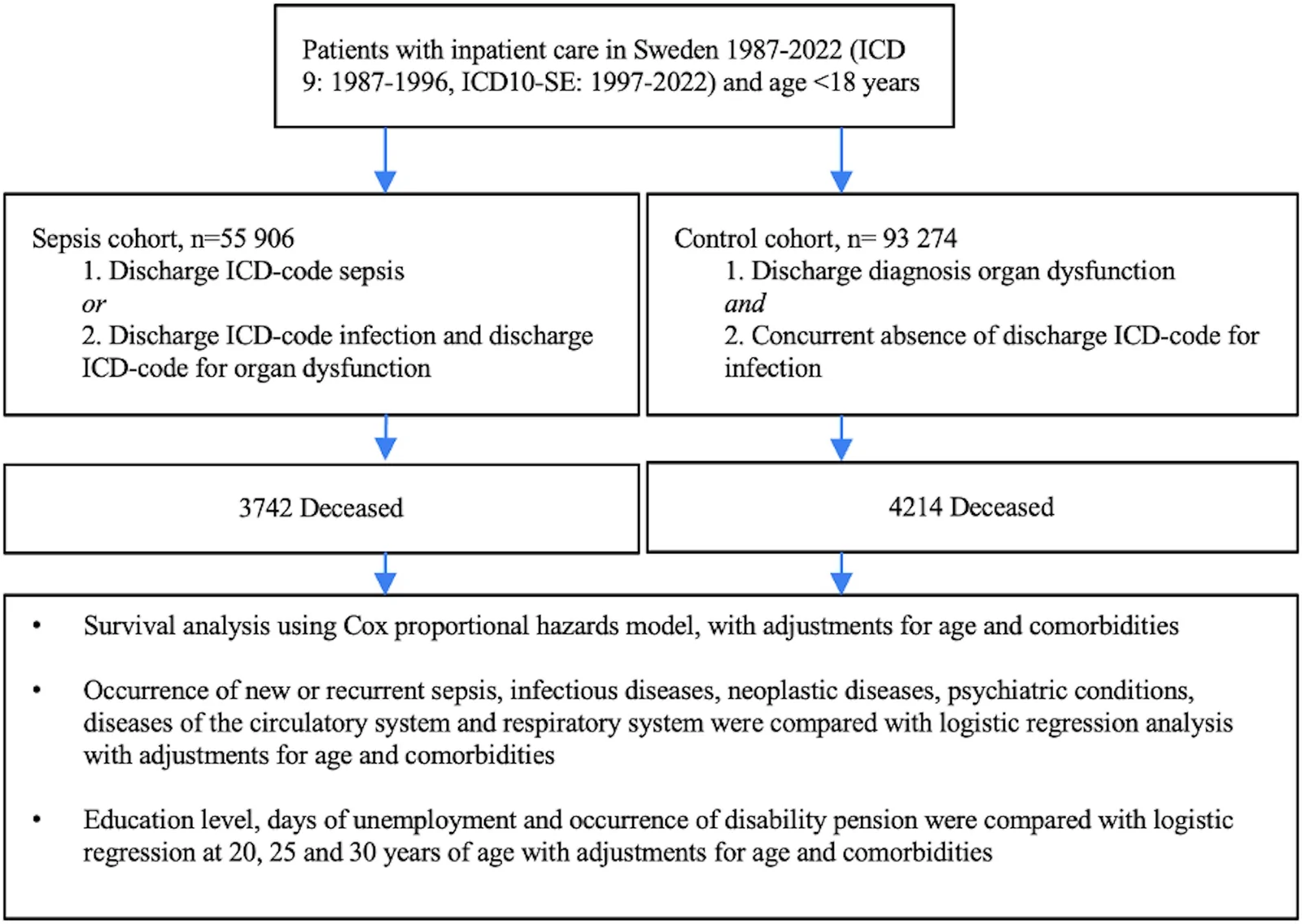
Flow diagram shows the inclusion criteria of the study, the number of pediatric hospitalizations in the sepsis cohort and the control cohort with organ dysfunction, the numbers of deceased in each group, and a list of outcomes and comparative analyses performed. *ICD*, *International Classification of Diseases*; *ICD-9*, *International Classification of Diseases, Ninth Revision, Swedish Edition*; *ICD-10-SE*, *International Classification of Diseases and Related Health Problems, Tenth Revision, Swedish Edition*.

**Table 1.** Baseline Values and Comorbidities by Index Hospitalization

	Median [IQR] or No. (%)		
Variable	Overall (n = 149 180)	Control Cohort (n = 93 274)	Sepsis Cohort (n = 55 906)
Age, y	0 [0–0]	0 [0–0]	0 [0–1]
0–1	120 346 (81)	78 639 (84)	41 707 (75)
1–7	15 490 (10)	7631 (8.2)	7859 (14)
7–13	5607 (3.8)	2889 (3.1)	2718 (4.9)
13–18	7727 (5.2)	4109 (4.4)	3618 (6.5)
Sex			
Male	86 078 (58)	53 569 (57)	32 509 (58)
Female	63 102 (42)	39 705 (43)	23 397 (42)
Neoplasms	3663 (2.5)	624 (0.7)	3039 (5.4)
Diseases of the respiratory system	3793 (2.5)	2239 (2.4)	1554 (2.8)
Congenital condition	5127 (3.4)	2903 (3.1)	2224 (4.0)
Hemiplegia, tetraplegia	612 (0.4)	309 (0.3)	303 (0.5)
Rheumatic disease	480 (0.3)	217 (0.2)	263 (0.5)
Diabetes	224 (0.2)	118 (0.1)	106 (0.2)
Moderate or severe kidney disease	315 (0.2)	161 (0.2)	154 (0.3)
Liver disease	272 (0.2)	110 (0.1)	162 (0.3)
Congestive heart failure or myocardial infarction	212 (0.1)	113 (0.1)	99 (0.2)
Cerebrovascular disease	193 (0.1)	101 (0.1)	92 (0.2)
No comorbidities	136 951 (92)	87 593 (94)	49 358 (88)
Follow-up, y			
All	13.3	12.8	14.1
Survivors only	14.3	13.6	15.4

The median follow-up time from inclusion to censoring or death was 13.3 years: 14.1 years in the sepsis cohort and 12.8 years in the control cohort. In the sepsis cohort, 6548 (12%) patients had preexisting comorbidities at inclusion, as compared with 5681 (6%) in the control cohort.

### Mortality

In the sepsis cohort, 1250 (2.2%), 2403 (4.4%), and 3110 (6.6%) patients were reported deceased at 30 days, 1 year, and 5 years after discharge, respectively, and 3742 (6.7%) during the entire follow-up. The numbers of reported deceased in the control cohort were 1853 (2.0%), 2728 (3.0%), and 3286 (4.4%) at 30 days, 1 year, and 5 years and 4214 (4.5%) during total follow-up.

The crude HR was 1.46 (95% CI, 1.40–1.53) in the sepsis cohort and remained elevated at 1.17 (95% CI, 1.11–1.22) after adjustments (Supplementary Figure 1).

Overall, the excess mortality of the sepsis cohort occurred within the first 5 years after discharge (Figure 2). Among the patients surviving the first 5 years after inclusion, the hazard was lower in the sepsis group, with a crude HR of 1.06 (95% CI, .96–1.18) and an adjusted HR of 0.87 (95% CI, .78–.97).

**Figure 2.**
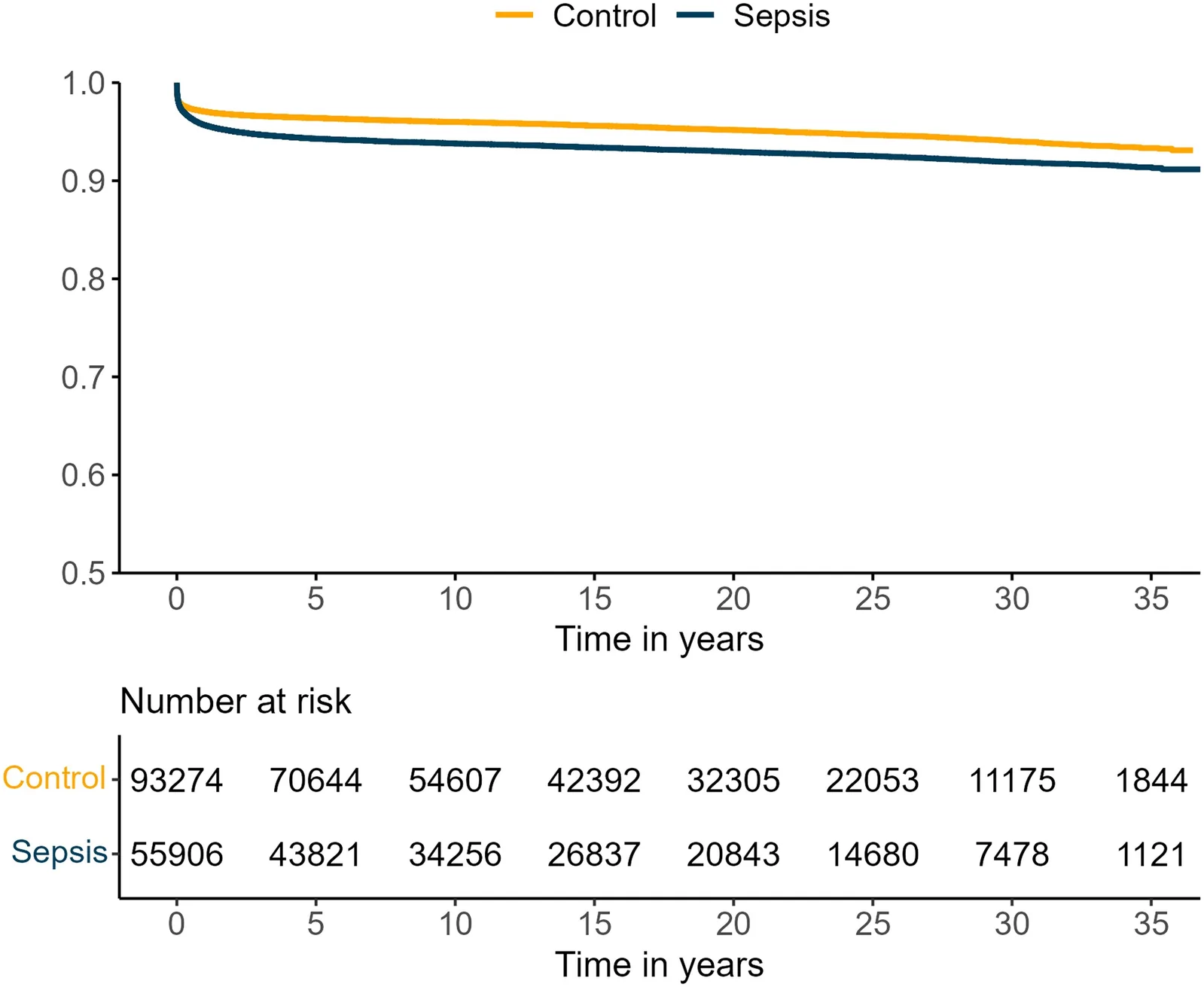
Crude survival plot compares the pediatric sepsis cohort and the pediatric control cohort with organ dysfunction, showing mortality 0 to 35 years after discharge.

In the sensitivity analyses, patients in the sepsis cohort defined by the *ICD-9* had an adjusted HR of 0.88 (95% CI, .81–.95); those defined by the *ICD-10-SE*, an adjusted HR of 1.36 (95% CI, 1.28–1.44); and those with available outpatient data, an adjusted HR of 1.41 (95% CI, 1.32–1.50; Supplementary Table 7). In the age-stratified sensitivity analysis, the strata of patients <7 years old had elevated HRs and the strata ≥7 years did not. Mortality data on the age-stratified sensitivity analysis are available in Supplementary Table 6.

### Morbidity

The number of patients with occurrence of sepsis at any time point during total follow-up was 5715 (10%) in the sepsis cohort and 3410 (3.7%) in the control cohort.

Within the first 3 months after discharge, the adjusted odds ratio (OR) for recurrent sepsis was 4.44 (95% CI, 4.09–4.81; Table 2). The adjusted OR was 1.56 (95% CI, 1.41–1.72) at 3 months to 1 year after discharge and 1.30 (95% CI, 1.18–1.44) at 1 to 5 years after discharge.

**Table 2.** Logistic Regression: Morbidity Outcome 0–5 Years After Discharge

	No. (%)			Odds Ratio (95% CI)	
Variable	Overall (n = 94 258)	Control Cohort (n = 60 403)	Sepsis Cohort (n = 33 855)	Crude	Adjusted^a^
New diagnosis within 3 mo of discharge after index hospitalization					
Sepsis	3988 (2.7)	817 (0.9)	3171 (5.8)	6.87 (6.36–7.43)	4.44 (4.09–4.81)
Infectious diseases	15 117 (10)	8584 (9.4)	6533 (12)	1.32 (1.27–1.36)	1.19 (1.15–1.23)
Neoplasms	893 (0.6)	488 (0.5)	405 (0.7)	1.40 (1.23–1.60)	0.93 (.80–1.07)
Mental and behavioral disorders	943 (0.6)	640 (0.7)	303 (0.6)	0.80 (0.69–.91)	0.68 (0.59–.79)
Diseases of the circulatory system	4153 (2.8)	2732 (3.0)	1421 (2.6)	0.87 (.82–.93)	0.83 (.77–.88)
Diseases of the respiratory system	2365 (1.6)	1382 (1.5)	983 (1.8)	1.20 (1.11–1.30)	1.11 (1.02–1.21)
New diagnosis 3–12 mo after discharge after index hospitalization					
Sepsis	1829 (1.3)	752 (0.8)	1077 (2.0)	2.44 (2.22–2.68)	1.56 (1.41–1.72)
Infectious diseases	22 326 (16)	13 736 (15)	8590 (16)	1.06 (1.03–1.10)	1.06 (1.03–1.09)
Neoplasms	1257 (0.9)	694 (0.8)	563 (1.1)	1.37 (1.23–1.53)	0.91 (.80–1.03)
Mental and behavioral disorders	2140 (1.5)	1384 (1.5)	756 (1.4)	0.92 (0.84–1.00)	0.83 (0.76–.91)
Diseases of the circulatory system	4561 (3.2)	2914 (3.2)	1647 (3.1)	0.95 (.89–1.01)	0.90 (.85–.96)
Diseases of the respiratory system	6606 (4.6)	3854 (4.3)	2752 (5.2)	1.21 (1.15–1.28)	1.24 (1.17–1.30)
New diagnosis 1–5 y after discharge after index hospitalization					
Sepsis	1730 (1.2)	891 (1.0)	839 (1.6)	1.60 (1.45–1.76)	1.30 (1.18–1.44)
Infectious diseases	23 344 (17)	14 915 (17)	8429 (16)	0.94 (.92–.97)	0.97 (.94–1.00)
Neoplasms	1985 (1.4)	1002 (1.2)	983 (1.9)	1.67 (1.53–1.82)	1.09 (0.99–1.20)
Mental and behavioral disorders	7193 (5.2)	4422 (5.1)	2771 (5.4)	1.06 (1.01–1.11)	0.98 (.93–1.03)
Diseases of the circulatory system	9171 (6.6)	5653 (6.5)	3518 (6.8)	1.05 (1.01–1.10)	1.00 (0.96–1.05)
Diseases of the respiratory system	18 334 (13)	11 472 (13)	6862 (13)	1.01 (.98–1.04)	1.06 (1.02–1.09)

^a^Adjusted for age and comorbidities.

After adjustments, acquiring new infectious disease was slightly more common in the sepsis cohort during the first 3 months after discharge, with an adjusted of OR 1.19 (95% CI, 1.15–1.23), although this association decreased with follow-up time. The adjusted OR was 1.06 (95% CI, 1.03–1.09) at 3 to 12 months after discharge and was not elevated at 1 to 5 years after discharge, with an OR of 0.97 (95% CI, .94–1.00).

The OR for acquiring neoplastic disease was elevated in the crude analysis (Table 2) but not after adjustments. The adjusted ORs for new psychiatric conditions were 0.68 (95% CI, .59–.79), 0.83 (95% CI, .76–.91), and 0.98 (95% CI, .93–1.03) at 0 to 3 months, 3 to 12 months, and 1 to 5 years after discharge, respectively.

It was slightly less common to acquire new disease of the circulatory system in the sepsis cohort in the first 3 months after follow-up, and the adjusted ORs were 0.83 (95% CI, .77–.88), 0.90 (95% CI, .85–.96), and 1.00 (95% CI, .96–1.05) for 0 to 3 months, 3 to 12 months, and 1 to 5 years after discharge, respectively. The adjusted ORs for new respiratory disease after discharge were 1.11 (95% CI, 1.02–1.21), 1.24 (95% CI, 1.17–1.30), and 1.06 (95% CI, 1.02–1.09) at 0 to 3 months, 3 to 12 months, and 1 to 5 years after discharge, respectively.

The results from the sensitivity analysis of morbidity stratified by 0 to <1 and 1 to <18 years of age are displayed in Supplementary Table 8. The competing risk analysis is presented in Supplementary Table 5.

#### Cause of Death

Of the 3742 deaths in the sepsis cohort, 1386 (37%) were related to sepsis, either subsequent or per index hospitalization, as opposed to 500 (12%) of 4214 in the control cohort. Further data on causes of death are presented in Table 3.

**Table 3.** Causes of Death by *International Classification of Diseases* Chapters

	No. (%)		
Variable	Overall (n = 7956)	Control Cohort (n = 4214)	Sepsis Cohort (n = 3742)
Sepsis	1886 (24)	500 (12)	1386 (37)
Organ dysfunction	4033 (51)	2241 (53)	1792 (48)
Infectious diseases	1746 (22)	651 (15)	1095 (29)
Neoplasms	1328 (17)	319 (7.6)	1009 (27)
Diseases of the blood and blood-forming organs	418 (5.3)	132 (3.1)	286 (7.6)
Endocrine, nutritional and metabolic diseases	540 (6.8)	319 (7.6)	221 (5.9)
Mental and behavioral disorders	338 (4.2)	196 (4.7)	142 (3.8)
Diseases of the			
Nervous system	1508 (19)	822 (20)	686 (18)
Eye and adnexa	26 (0.3)	13 (0.3)	13 (0.3)
Ear and mastoid process	13 (0.2)	9 (0.2)	4 (0.1)
Circulatory system	2513 (32)	1322 (31)	1191 (32)
Respiratory system	1030 (13)	496 (12)	534 (14)
Digestive system	640 (8.0)	271 (6.4)	369 (9.9)
Skin and subcutaneous tissue	18 (0.2)	8 (0.2)	10 (0.3)
Musculoskeletal system and connective tissue	190 (2.4)	108 (2.6)	82 (2.2)
Genitourinary system	311 (3.9)	118 (2.8)	193 (5.2)
R codes^a^	2488 (31)	1281 (30)	1207 (32)
Other	5067 (64)	2961 (70)	2106 (56)

All causes of death for each individual included.

^a^Symptoms, signs, and abnormal clinical and laboratory findings not elsewhere classified.

#### Socioeconomic Factors

The sepsis cohort had an elevated adjusted OR for achieving a higher level of education, which remained similar at 20, 25, and 30 years of age (Supplementary Table 3). Occurrence of unemployment was similar in the sepsis cohort and the control cohort after adjustments (Supplementary Table 9). Disability pension was more common in the control cohort, with adjusted ORs of 0.85, 0.80, and 0.75 at 20, 25, and 30 years, respectively (Supplementary Table 10).

## DISCUSSION

In this nationwide cohort study, we demonstrate excess mortality in pediatric patients with sepsis in the first 5 years after discharge as compared with a control cohort with organ dysfunction. Furthermore, the sepsis cohort had a clear overrepresentation of a new sepsis event during follow-up that remained during the first 5 years, with ORs ranging from 4.44 to 1.30. Our data show that the first year after discharge is where the differences in morbidity between the cohorts are most evident. Beyond sepsis, infectious disease and respiratory disease were more common in the sepsis cohort within the first year. To our knowledge, this is the longest follow-up after pediatric sepsis and the first to provide long-term data on morbidity.

Our study has limitations. The implicit criteria method has been sparsely validated in children, with varying results and positive predictive values ranging from 22% to 87% [15, 24, 25]. In adults, sensitivity of explicit *ICD* codes alone has been shown to be insufficient [16, 26], as recently demonstrated in Sweden [17]. The combination of explicit and implicit codes aims to adjust for misclassification and is likely to add sensitivity [16, 27] to the cost of lower specificity. This could entail misclassification bias and give rise to underestimates of the true long-term effects of sepsis.

More generally, using administrative data from health registers conveys a risk of misclassification, as data are not originally recorded for the purpose of research. Also, the definitions and understanding of sepsis have evolved since 1987 [28–31].

Moreover, as data detail did not allow for adjustment for gestational age at birth, we identify prematurity as a potential unmeasured confounder that could influence the outcomes. However, comorbidities related to prematurity, such as lung disease and disability, are sought to be accounted for using Charlson Comorbidity Index. Due to granularity of the data, we could not separate out neonatal hospitalizations from older infant hospitalizations; thus, the age group from 0 to 1 year could hide differences in outcomes between these clinically different groups. Furthermore, the sepsis cohort could not be divided by sepsis severity, as severity scoring and biomarkers were not available for analysis; hence, implications of disease severity on long-term outcome could not be explored.

Another limitation is that the secondary outcomes, the socioeconomic in particular, must be interpreted cautiously, as no adjustments were made for multiple hypothesis testing and the regression models could not be adjusted for socioeconomic factors at baseline. Long-term socioeconomic outcome could be affected by preexisting socioeconomic differences. Multiple-testing adjustment was not performed as the analysis consisted of 1 primary and multiple secondary outcomes.

Last, the risk of residual confounding should be acknowledged. The control cohort and sepsis cohort are different in nature, and the Charlson Comorbidity Index may not have been able to sufficiently capture differences in underlying health status. Also, factors such as immunodeficiency, socioeconomic background, and ethnicity, which were not available for our analysis, could constitute residual confounders.

The markedly differing HRs of the *ICD-9* and *ICD-10-SE* periods in the sensitivity analysis are noteworthy and may be a result of changing coding practices, *ICD-10-SE* updates, and the absence of comorbidity data for adjustments in the older data. We included both periods, although we believe that *ICD-10-SE* data are likely to be more reliable. Most of our data are contained within *ICD-10-SE*, in which the adjusted HR was 1.36 in comparison with the overall adjusted HR of 1.17.

The study investigates long-term disease burden after sepsis in relation to hospitalized children with organ dysfunction, not healthy individuals, and thus likely underestimates the apparent disease burden as opposed to comparing with the general pediatric population. However, comparing with a control cohort from the general population could have led to overestimates, as the groups may not be comparable with respect to underlying health status.

Our data on mortality are in line with, to our knowledge, the only study with data on long-term mortality after pediatric sepsis [32], which suggests the most frequent occurrence of late death at 2 years after discharge and late cases of death up to 10 years after discharge.

Previous studies have shown an association between pediatric sepsis and new or progressive health conditions, although the association has been limited to certain conditions and the months following sepsis [10, 11, 33]. Sankar et al found physical health impairment after severe sepsis in children within the first 12 months [34]. Without previously published data on morbidity beyond 1 year, our study contributes with important findings by emphasizing the value of postsepsis care or vigilance with a long perspective beyond the first 12 months and with additional focus on the first years following sepsis. This is in accordance with postsepsis studies in adults, which demonstrate a similar pattern but different long-term morbidity [7, 8, 35, 36].

We were unable to discriminate intensive care patients due to limitations of the data set. With the inclusion of neonatal patients and the fact that we did not access outpatient care data until 2001, this may also explain the larger proportion of patients with sepsis without previous chronic disease (88%) as compared with other studies [34, 37, 38].

Our study population with sepsis is young vs other studies of pediatric sepsis and severe sepsis [33, 34, 37, 39]. Their varying inclusion methods and our choice to include neonatal patients likely account for some of this difference.

We cannot conclude what are the drivers behind the higher mortality and morbidity in the sepsis cohort. Further studies are needed to find out more about mechanisms of excess mortality. As we did not find any overrepresentation of new neoplastic disease after sepsis in our morbidity analysis, we believe that the higher frequency of neoplastic disease as the cause of death in the sepsis cohort is likely due to higher unadjusted prevalence of malignancy at baseline. However, it is notable that neoplastic disease was recently found to be more common in adult survivors of sepsis than in the general population [40].

A paradoxical result in the age-stratified sensitivity analysis is the long-term hazard rate after sepsis hospitalizations of the age group of 13 to 18 years (Supplementary Table 5), which is lower than the control group, in contrast with other age categories. The reasons cannot be determined from the data, although possible explanations could be that the vulnerable children most susceptible to sepsis account for a larger share of the excess mortality and may have already been afflicted by the age of 13 years or that the control group of 13 to 18 years encircles a more vulnerable population of children with worse prognosis. This could also explain the lower HR in the 1- to 18-year age group, for which 1.00 is contained within the 95% CI. Moreover, the results of the age-stratified sensitivity analyses are broadly consistent with the overall results, although they suggest that the older children and adolescents in this study either encircle different populations or are less prone to late morbidity and mortality than the younger children. Last, the results from the competing risk analysis did not display notable differences from the main results, suggesting little impact of the higher mortality in the sepsis cohort.

The major strengths of the study are the long-term and extensive follow-up as well as the nationwide data, which improve generalizability. We performed adjustments and a comprehensive number of subgroup and sensitivity analyses to assess the effect of heterogeneity and strengthen the robustness of the results.

## CONCLUSIONS

Children surviving sepsis have a sustained disease burden for a long time after discharge, although the first years seem to be most affected. The causes behind this presumed vulnerability remain to be elucidated. The findings call for further studies of postsepsis prognosis in children and enhanced follow-up and vigilance during the years after pediatric sepsis to prevent disease and death.

## Supplementary Material

ofag660_Supplementary_Data

## Supplementary Data

Supplementary materials are available at *Open Forum Infectious Diseases* online. Consisting of data provided by the authors to benefit the reader, the posted materials are not copyedited and are the sole responsibility of the authors, so questions or comments should be addressed to the corresponding author.
