# Supplementary material for: Long-Term Mortality and Morbidity After Pediatric Sepsis
Source: Open Forum Infect Dis. 2026 Oct 7;13(10):ofag660. doi: 10.1093/ofid/ofag660 (PMC13678215; doi:10.1093/ofid/ofag660)
Supplement: Supplementary file 1 [file ofag660_supplementary_data.docx]

# Supplementary Information, Long-term Mortality and Morbidity After Pediatric Sepsis

Table of Contents

[eFigure 1, Survival Plot, Adjusted	2](#_Toc239688849)

[eTable 1, Table of Codes for Explicit Sepsis Criteria, Infection Criteria and Organ Dysfunction Criteria in Swedish Edtitions of ICD 9 and 10	3](#_Toc239688850)

[eTable 2, Table of ICD-codes in ICD-10-SE and ICD-9 SE Used for the Outcome Analysis and Grouped by Chapter	25](#_Toc239688851)

[eTable 3, Educational Status in Sepsis Cohort at 20, 25 and 30 years of age, Stepwise Logistic Regression Analysis	53](#_Toc239688852)

[eTable 4, Charlson Comorbidity Index, Swedish Adaption	54](#_Toc239688853)

[eTable 5, Competing Risk Analysis of Mordbidity in Sepsis Cohort with Fine-Gray Subdistribution Hazard Model, with Death as Competing Risk	67](#_Toc239688854)

[eTable 6, Age Stratification of Overall Survival, Cox Regression Analysis	68](#_Toc239688855)

[eTable 7, Sensitivity Analyses of Overall Survival, Cox Regression Analysis	69](#_Toc239688856)

[eTable 8, Sensitivity Analysis of Morbidity Outcome, 0-<1 Years and 1-<18 Years, Logistic Regression, 0-5 Years After Discharge	70](#_Toc239688857)

[eTable 9, Unemployment, Stepwise Logistic Regression Analysis	73](#_Toc239688858)

[eTable 10, Occurrence of Disability Pension, Logistic Regression Analysis	74](#_Toc239688859)

## eFigure 1, Survival Plot, Adjusted

eFigure 1. Survival plot comparing the pediatric sepsis cohort and the pediatric control cohort with organ dysfunction, showing mortality 0 to 35 years after discharge. The figure is adjusted for age and comorbidities.

Alt text: Adjusted survival plot, depicting survival of the pediatric sepsis cohort and the control cohort with organ dysfunction with separate graphs, 0-35 years after discharge. The graphs depict a distinct initial drop in survival of both cohort curves within the first years whereafter the survival curves slowly divert during the subsequent follow up time as survival in the sepsis cohort is lower.

## eTable 1, Table of Codes for Explicit Sepsis Criteria, Infection Criteria and Organ Dysfunction Criteria in Swedish Edtitions of ICD 9 and 10

|  |  | **ICD-10-SE** |  | **ICD-9 SE (Klassifikation av sjukdomar 1987), translation from ICD-10-SE** | |
| --- | --- | --- | --- | --- | --- |
| **Explicit sepsis codes** |  | A021 | Salmonella sepsis | 003B | Salmonella Septicemia |
|  |  | A200 | Bubonic plague | 020. | Plague |
|  |  | A207 | Septicaemic plague |  |  |
|  |  | A217 | Generalized tularaemia | 021X | Tularaemia |
|  |  | A227 | Anthrax sepsis | 022 | Anthrax |
|  |  | A241 | Acute or fulminating melioidosis | 025 | Meloidosis |
|  |  | A267 | Erysipelothrix sepsis | 027B | Erysipelothrix |
|  |  | A282 | Extraintestinal yersiniosis |  |  |
|  |  | A327 | Listerial sepsis | 027A | Listeriosis |
|  |  | A391 | Waterhouse-Friderichsen syndrome | 036D | Waterhouse-Friderichsen syndrome caused by meningococcal infection |
|  |  | A392 | Acute meningococcal sepsis | 036C | Meningococcaemia, Meningococcal sepsis |
|  |  | A393 | Chronic meningococcemia |  |  |
|  |  | A394 | Meningococcaemia, unspecified |  |  |
|  |  | A40 | Streptococcal sepsis | 038A | Streptokocksepticaemia |
|  |  | A41 | Other sepsis | 038. | Septichaemia |
|  |  | A427 | Actinomycotic sepsis | 039X | Actinomycosis |
|  |  | A483 | Toxic shock syndrome |  |  |
|  |  | A499 | Bacterial infection, unspecified | 041X | Bacterial infection, classified elsewhere and with non-specified location |
|  |  | A548 | Other gonococcal infections | 098W | Gonococcal infection with other location, gonococcaemia/endocarditis/keratosis/meningitis/peritonitis |
|  |  | B007 | Disseminated herpesviral disease |  |  |
|  |  | B376 | Candidal endocarditis | 112W | Candidainfection with other localization Candidaendocarditis Candidainfection in esofagus |
|  |  | B377 | Candidal sepsis | 112F | Disseminated candidal infection Systemic candidiasis |
|  |  | B49 | Unspecified mycosis | 117X | Other and unspecified mycosis |
|  |  | O753 | Other infection or sepsis during labour | 659D | General infection during labour, Sepsis during labour |
|  |  | O85 | Other puerperal infections | 670. | Severe infection during puerperium, Infectio gravior in puerperio, Puerperal pelvic peritonitis/endometritis/fever/peritonitis |
|  |  |  |  |  |  |
|  |  | R651 | Sepsis according to Sepsis-3-criteria |  |  |
|  |  | R572 | Sepsic chock according to Sepsis-3-criteria |  |  |
|  |  |  |  |  |  |
|  |  | P36 | Bacterial sepsis in newborn | 771W | Other infections specific to the perinatal period, Septichaemia neonatorum |
|  |  |  |  |  |  |
| **Infections** | **Respiratory infections** | J01 | Acute sinusitis | 461 | Acute sinusitis |
|  |  | J02 | Acute pharyngitis | 462 | Acute pharyngitis |
|  |  | J03 | Acute tonsillitis | 463 | Acute tonsillitis |
|  |  | J04 | Acute laryngitis and tracheitis | 464 | Acute laryngitis and tracheitis |
|  |  | J06 | Acute upper respiratory infections of multiple and unspecified sites | 465 | Acute infection of upper respiratory tract, multiple or unspecified localization |
|  |  | J05 | Acute obstructive laryngitis [croup] and epiglottitis | 464C-464E | Acute laryngotracheitis/epiglottitis, pseudocroup |
|  |  | J09 | Influenza due to identified zoonotic or pandemic influenza virus | 487 | Influenza |
|  |  | J10 | Influenza due to identified seasonal influenza virus | 487 | Influenza |
|  |  | J11 | Influenza, virus not identified | 487 | Influenza |
|  |  | J12 | Viral pneumonia | 480 | Viral pneumonia |
|  |  | J13-18 | Pneumonia J13 Pneumonia due to Streptococcus pneumoniae J14 Pneumonia due to Haemophilus influenzae J15 Bacterial pneumonia, not elsewhere classified J16 Pneumonia due to other infectious organisms, not elsewhere classified J17 Pneumonia in diseases classified elsewhere J18 Pneumonia, organism unspecified | 481-486 | 481 Pneumonia caused by Pneumococci, Lobar pneumonia, non-specified microorganism 482 Other bacterial pneumonia 483 Pneumonia caused by other specified microorganisms, mycoplasma pneumonia 484 Pneumonia in other infectious diseases classified elsewhere 485 Bronchopneumonia 486 Pneumonia caused by non-specified microorganism |
|  |  | J13 | Pneumonia due to Streptococcus pneumoniae |  |  |
|  |  | J14 | Pneumonia due to Haemophilus influenzae |  |  |
|  |  | J15 | Bacterial pneumonia, not elsewhere classified |  |  |
|  |  | J16 | Pneumonia due to other infectious organisms, not elsewhere classified | |  |
|  |  | J17 | Pneumonia in diseases classified elsewhere |  |  |
|  |  | J18 | Pneumonia, organism unspecified |  |  |
|  |  | J20 | Acute bronchitis | 466 | Acute bronchitis and bronchiolitis |
|  |  | J21 | Acute bronchiolitis | 466B | Acute bronchiolitis |
|  |  | J22 | Unspecified acute lower respiratory infection | 466A | Acute bronchitis |
|  |  | J440 | Chronic obstructive pulmonary disease with acute lower respiratory infection | |  |
|  |  | J441 | Chronic obstructive pulmonary disease with acute exacerbation, unspecified | |  |
|  |  | J86 | Pleural empyema | 510 | Empyema |
|  |  | J85 | Abscess of lung and mediastinum | 513 | Abscess of lung and mediastinum |
|  |  | A15 | Respiratory tuberculosis, bacteriologically or histologically confirmed | 010-012 | 010 Primary tuberculosis infection 011 Lungtuberkulos 012 Annan tuberkulos i andningsorganen |
|  |  | A16 | Respiratory tuberculosis, not confirmed bacteriologically or histologically | |  |
|  |  |  |  |  |  |
|  |  | A36 | Diphtheria | 032. | Diphtheria |
|  |  | A37 | Whooping cough | 033. | Whooping cough |
|  |  | B38 | Coccidioidomycosis | 114 | Coccidioidomycosis |
|  |  | B39 | Histoplasmosis | 115 | Histoplasmosis |
|  |  | P23 | Congenital pneumonia | 770A | Congenital pneumonia |
|  |  | P240 | Neonatal aspiration of meconium | 770B | Neonatal aspiration of meconium |
|  |  | P248 | Other neonatal aspiration syndromes | 770B | Neonatal aspiration of meconium |
|  |  | P249 | Neonatal aspiration syndrome, unspecified | 770B | Neonatal aspiration of meconium |
|  |  |  |  |  |  |
|  | **Abdominal infections** | A00 | Cholera | 001. | Cholera |
|  |  | A01 | Typhoid and paratyphoid fevers | 002. | Typhoid and paratyphoid fevers |
|  |  | A02 | Other salmonella infections | 003. | Other salmonella infections |
|  |  | A03 | Shigellosis | 004. | Shigellosis |
|  |  | A04 | Other bacterial intestinal infections | 008. | Other bacterial intestinal infections |
|  |  | A05 | Other bacterial foodborne intoxications, not elsewhere classified | 005. | Other bacterial foodborne intoxications |
|  |  | A06 | Amoebiasis | 006. | Amoebiasis |
|  |  | A07 | Other protozoal intestinal diseases | 007. | Other protozoal intestinal diseases |
|  |  | A08 | Viral and other specified intestinal infections | 008. | Other bacterial intestinal infections |
|  |  | A09 | Other gastroenteritis and colitis of infectious and unspecified origin | 009. | Ill-defined intestinal infections |
|  |  | K35 | Acute appendicitis | 540 | Acute appendicitis |
|  |  | K37 | Unspecified appendicitis | 541 | Unspecified appendicitis |
|  |  | K36 | Other appendicitis | 542 | Other appendicitis |
|  |  | K570-K575 | Diverticular disease of small intestine with perforation and abscess | 562A | Diverticula of small intestine |
|  |  |  |  |  |  |
|  |  | K571 | Diverticular disease of small intestine without perforation and abscess | 562A | Diverticula of small intestine |
|  |  |  |  |  |  |
|  |  | K572 | Diverticular disease of large intestine with perforation and abscess | 562B | Diverticula of colon |
|  |  | K573 | Diverticular disease of large intestine without perforation or abscess | 562B | Diverticula of colon |
|  |  |  |  |  |  |
|  |  |  |  |  |  |
|  |  | K574 | Diverticular disease of both small and large intestine with perforation and abscess | | |
|  |  |  |  |  |  |
|  |  | K575 | Diverticular disease of both small and large intestine without perforation or abscess | | |
|  |  | U071 | Covid-19, virus identified |  |  |
|  |  | U072 | Covid-19, virus not identified |  |  |
|  |  | U109 |  |  |  |
|  |  |  |  |  |  |
|  |  |  |  |  |  |
|  |  | K61 | Abscess of anal and rectal regions | 566 | Abscess of anal and rectal regions |
|  |  | K65 | Peritonitis | 567 | Peritonitis |
|  |  | K67 | Disorders of peritoneum in infectious diseases classified elsewhere | |  |
|  |  | K630 | Abscess of intestine | 569F | Abscess of intestine |
|  |  | K631 | Perforation of intestine (nontraumatic) | 530E | Perforation of esophagus (nontraumatic) |
|  |  |  |  | 531B | Acute ulcer of ventricle with perforation |
|  |  |  |  | 531C | Acute ulcer of ventricle with perforation and bleeding |
|  |  |  |  | 531F | Chronic or not specified ulcer of ventricle with perforation |
|  |  |  |  | 531G | Chronic or not specified ulcer of ventricle with perforation and bleeding |
|  |  |  |  | 532B | Acute ulcer of duodenum with perforation |
|  |  |  |  | 532C | Acute ulcer of duodenum with perforation and bleeding |
|  |  |  |  | 532F | Chronic or not specified ulcer of duodenum with perforation |
|  |  |  |  | 532G | Chronic or not specified ulcer of duodenum with perforation and bleeding |
|  |  |  |  | 533B | Acute ulcer of ventricle or duodenum with perforation |
|  |  |  |  | 533C | Acute ulcer of ventricle or duodenum with perforation and bleeding |
|  |  |  |  | 533F | Chronic or not specified ulcer of ventricle or duodenum with perforation |
|  |  |  |  | 533G | Chronic or not specified ulcer of ventricle or duodenum with perforation and bleeding |
|  |  |  |  | 534B | Acute ulcer of ventricle with perforation, relapse after surgery |
|  |  |  |  | 534C | Acute ulcer of ventricle with perforation and bleeding, relapse after surgery |
|  |  |  |  | 534F | Chronic or not specified ulcer of ventricle with perforation, relapse after surgery |
|  |  |  |  | 534G | Chronic or not specified ulcer of ventricle with perforation and bleeding, relapse after surgery |
|  |  | K750 | Abscess of liver | 572A | Abscess of liver |
|  |  | K751 | Phlebitis of portal vein | 572B | Pylephlebitis |
|  |  | K810 | Acute cholecystitis | 575A | Cholecystitis |
|  |  | K770 | Liver disorders in infectious and parasitic diseases classified elsewhere | |  |
|  |  |  |  |  |  |
|  |  | P38 | Omphalitis of newborn with or without mild haemorrhage | 771E | Omphalitis of newborn |
|  |  | P77 | Necrotizing enterocolitis of fetus and newborn | 777F | Necrotizing enterocolitis of fetus and newborn |
|  |  | P781 | Other neonatal peritonitis |  |  |
|  |  |  |  |  |  |
|  | **Wound and soft tissue infections** | A46 | Erysipelas | 035. | Erysipelas |
|  |  | B47 | Mycetoma | 117E | Mycotic Mycetoma |
|  |  | L03 | Cellulitis | 681 | Cellulitis |
|  |  |  |  | 682 | Other cellulitis |
|  |  | L04 | Acute lymphadenitis | 683 | Acute lymphadenitis |
|  |  | L08 | Other local infections of skin and subcutaneous tissue | 686 | Other local infections of skin and subcutaneous tissue |
|  |  | L05 | Pilonidal cyst | 685A | Pilonidal cyst with abscess |
|  |  | B00 | Herpesviral [herpes simplex] infections | 054. | Herpesviral [herpes simplex] infections |
|  |  | B07 | Viral warts | 078B | Viral warts |
|  |  | B08 | Other viral infections characterized by skin and mucous membrane lesions, not elsewhere classified | 057. | Other skin lesions caused by virus |
|  |  |  |  | 051. | Cowpox |
|  |  |  |  | 074A | Herpangina |
|  |  |  |  | 078A | Molluscum contagiosum |
|  |  | B09 | Unspecified viral infection characterized by skin and mucous membrane lesions | | |
|  |  | H050 | Acute inflammation of orbit | 376A | Acute inflammation of orbit |
|  |  | H602 | Malignant otitis externa | 380B | Infectious otitis externa |
|  |  | H700 | Acute mastoiditis | 383A | Acute mastoiditis |
|  |  | J36 | Peritonsillar abscess | 475 | Peritonsillar abscess |
|  |  | J390 | Retropharyngeal and parapharyngeal abscess | 478C | Other diseases of pharynx not elsewhere classified |
|  |  | J391 | Other abscess of pharynx | 478C | Other diseases of pharynx not elsewhere classified |
|  |  | L02 | Cutaneous abscess, furuncle and carbuncle | 680 | Cutaneous furuncle and carbuncle |
|  |  |  |  |  |  |
|  | **Genitourinary infections** | N10 | Acute tubulo-interstitial nephritis | 590B | Acute pyelonephritis and pyonephrosis |
|  |  | N151 | Renal and perinephric abscess | 590C | Renal and perinephric abscess |
|  |  | N159 | Renal tubulo-interstitial disease, unspecified | 590X | Kidney infection, unspecified |
|  |  | N34 | Urethritis and urethral syndrome | 597 | Non-venereal urethritis and urethral syndrome |
|  |  | N30 | Cystitis | 595 | Cystitis |
|  |  | N390 | Urinary tract infection, site not specified | 599A | Urinary tract infection, site not specified |
|  |  | N41 | Inflammatory diseases of prostate | 601A | Acute prostatitis |
|  |  |  |  | 601C | Abscess in prostate |
|  |  |  |  | 601D | Prostatocystitis |
|  |  | N45 | Orchitis and epididymitis | 604 | Orchitis and epididymitis |
|  |  | N482 | Other inflammatory disorders of penis | 607C | Other inflammatory disorders of penis / Cavernitis |
|  |  | N49 | Inflammatory disorders of male genital organs, not elsewhere classified | 608E | Inflammatory disorders of male genital organs |
|  |  | N70 | Salpingitis and oophoritis | 614A | Acute salpingitis and oophoritis |
|  |  |  |  | 614B | Chronic salpingitis and oophoritis |
|  |  |  |  | 614C | Salpingitis and oophoritis, not specified if acute, subacute or chronic |
|  |  | N71 | Inflammatory disease of uterus, except cervix | 615 | Inflammatory disease of uterus, except cervix |
|  |  | N72 | Inflammatory disease of cervix uteri | 616 | Inflammatory disease of cervix uteri, vagina and vulva |
|  |  | N73 | Other female pelvic inflammatory diseases | 614D-614F | 614D Acute parametritis and pelvic peritonitis 614E Chronic or unspecified parametritis and pelvic peritonitis 614F Acute or unspecified pevic peritonitis in female |
|  |  | N74 | Female pelvic inflammatory disorders in diseases classified elsewhere | |  |
|  |  | N75 | Diseases of Bartholin gland | 616D | Abscess in Bartholin gland |
|  |  | N76 | Other inflammation of vagina and vulva | 616 | Inflammatory disease of cervix uteri, vagina and vulva |
|  |  |  |  |  |  |
|  |  |  |  |  |  |
|  |  |  |  |  |  |
|  |  | N77 | Vulvovaginal ulceration and inflammation in diseases classified elsewhere | 616 | Inflammatory disease of cervix uteri, vagina and vulva |
|  |  | N61 | Inflammatory disorders of breast | 611A | Inflammatory disorders of breast |
|  |  | N980 | Infection associated with artificial insemination | 999D | Infection, sepsis or septicaemia caused by infusion, injection, transfusion or vaccination |
|  |  | A59 | Trichomoniasis | 131 | Trichomoniasis |
|  |  | A55 | Chlamydial lymphogranuloma (venereum) | 099B | Lymphogranuloma venereum |
|  |  | A56 | Other sexually transmitted chlamydial diseases | 078J | Chlamydial infektion in urogenital organs |
|  |  |  |  |  |  |
|  | **CNS infections** | A39 | Meningococcal infection | 036. | Meningococcal infection |
|  |  | G00 | Bacterial meningitis, not elsewhere classified | 320 | Bacterial meningitis |
|  |  | G01 | Meningitis in bacterial diseases classified elsewhere | 320H | Meningitis in bacterial diseases classified elsewhere |
|  |  | G02 | Meningitis in other infectious and parasitic diseases classified elsewhere | 321 | Meningitis caused by other microorganisms |
|  |  | G03 | Meningitis due to other and unspecified causes | 322A | 322A Non-purulent meningitis |
|  |  |  |  | 322B | 322B Eosinofil meningitis |
|  |  |  |  | 322X | 322X Meningit, unspecified |
|  |  | G04 | Encephalitis, myelitis and encephalomyelitis | 323W | Other specified encephalitis, myelitis and encephalomyelitis |
|  |  |  |  | 323X | Encephalitis, myelitis and encephalomyelitis, unspecified |
|  |  | G05 | Encephalitis, myelitis and encephalomyelitis in diseases classified elsewhere | |  |
|  |  | G06 | Intracranial and intraspinal abscess and granuloma | 324 | Intracranial and intraspinal abscess and granuloma |
|  |  | G07 | Intracranial and intraspinal abscess and granuloma in diseases classified elsewhere | 324 | Intracranial and intraspinal abscess and granuloma |
|  |  | G08 | Intracranial and intraspinal phlebitis and thrombophlebitis | 325 | Intracranial phlebitis and thrombophlebitis |
|  |  | A17 | Tuberculosis of nervous system | 013 | Tuberculosis of central nervous system and meninges |
|  |  | A81 | Atypical virus infections of central nervous system |  |  |
|  |  | A83 | Mosquito-borne viral encephalitis | 062. | Mosquito-borne viral encephalitis |
|  |  | A84 | Tick-borne viral encephalitis | 063. | Tick-borne viral encephalitis |
|  |  | A85 | Other viral encephalitis, not elsewhere classified | 064 | Viral encephalitis transmitted by other and unspecified arthropods |
|  |  |  |  | 048 | Other diseases of the central nervous system caused by enterovirus |
|  |  |  |  | 049 | Other diseases of the central nervous system not transmitted by arthropods |
|  |  | A86 | Unspecified viral encephalitis |  |  |
|  |  | A87 | Viral meningitis | 047. | Meningitis caused by enterovirus |
|  |  | A88 | Other viral infections of central nervous system, not elsewhere classified | |  |
|  |  | A89 | Unspecified viral infection of central nervous system |  |  |
|  |  |  |  |  |  |
|  | **Cardiovascular system** | I32 | Pericarditis in diseases classified elsewhere | 420 | Acute pericarditis |
|  |  | I33 | Acute and subacute endocarditis | 421A | Acute and subacute bacterial endocarditis |
|  |  | I39 | Endocarditis and heart valve disorders in diseases classified elsewhere | |  |
|  |  | I40 | Acute myocarditis |  |  |
|  |  | I41 | Myocarditis in diseases classified elsewhere |  |  |
|  |  | I80 | Thombosis, phlebitis and thrombophlebitis |  |  |
|  |  | I38 | Endocarditis, valve unspecified |  |  |
|  |  | I981 | Cardiovascular disorders in other infectious and parasitic diseases classified elsewhere | | |
|  |  |  |  |  |  |
|  | **Devise-Associated** | T826 | Infection and inflammatory reaction due to cardiac valve prosthesis | 996G | Infection and inflammatory reaction due to internal prosthesis , implants and grafts |
|  |  | T827 | Infection and inflammatory reaction due to other cardiac and vascular devices , implants and grafts | | |
|  |  | T835 | Infection and inflammatory reaction due to prosthetic device, implant and graft in urinary system | | |
|  |  | T836 | Infection and inflammatory reaction due to prosthetic device, implant and graft in genital tract | | |
|  |  | T845 | Infection and inflammatory reaction due to internal joint prosthesis | |  |
|  |  | T846 | Infection and inflammatory reaction due to internal fixation device [any site] | |  |
|  |  | T847 | Infection and inflammatory reaction due to other internal orthopaedic prosthetic devices, implants and grafts | | |
|  |  | T857 | Infection and inflammatory reaction due to other internal prosthetic devices, implants and grafts | | |
|  |  |  |  |  |  |
|  | **Other/unspecific** | A18 | Tuberculosis of other organs | 010-017 | 010 Primary tuberculosis infection 011 Pulmonary tuberculosis 012 Other tuberculosis in respiratory organ 013 Tuberculosis of the meninges and central nervous system 014 Tuberculosis of intestines, peritoneum, and mesenteric glands 015 Tuberculosis of bones and joints 016 Tuberculosis of genitourinary system 017 Tuberculosis of other organs |
|  |  | A19 | Miliary tuberculosis | 018. | Miliary tuberculosis |
|  |  | A20 | Plague | 020. | Plague |
|  |  | A21 | Tularaemia |  |  |
|  |  | A22 | Anthrax | 022. | Anthrax |
|  |  | A23 | Brucellosis | 023. | Brucellosis |
|  |  | A24 | Glanders and melioidosis | 024-025 | Glanders and melioidosis |
|  |  | A25 | Rat-bite fevers | 026. | Rat-bite fevers |
|  |  | A26 | Erysipeloid | 027B | Erysipeloid |
|  |  | A27 | Leptospirosis | 100 | Leptospirosis |
|  |  | A28 | Other zoonotic bacterial diseases, not elsewhere classified | 027C | Pasteurellosis |
|  |  |  |  | 078D | Cat-scratch disease |
|  |  |  |  | 027W | Other zoonotic bacterial diseases |
|  |  |  |  | 027X | Other zoonotic bacterial diseases, not specified |
|  |  | A32 | Listeriosis | 027A | Listeriosis |
|  |  | A38 | Scarlet fever | 034B | Scarlet fever |
|  |  | A42 | Actinomycosis | 039. | Actinomycosis |
|  |  | A43 | Nocardiosis |  |  |
|  |  | A44 | Bartonellosis | 088. | Other arthropod-borne diseases |
|  |  | A48 | Other bacterial diseases, not elsewhere classified | 040A | Gas gangrene |
|  |  |  |  | 482J | Legionella pneumonia |
|  |  | A49 | Bacterial infection of unspecified site | 041. | Bacterial infection classified elsewhere of unspecified site |
|  |  | A54 | Gonococcal infection | 098. | Gonococcal infection |
|  |  | A690 | Necrotizing ulcerative stomatitis | 101 | Vincent's angina |
|  |  |  |  | 528B | Cancrum oris |
|  |  | A691 | Other Vincent infections |  |  |
|  |  | A692 | Lyme disease | 104 | Other spirochetal infection |
|  |  | A698 | Other specified spirochaetal infections |  |  |
|  |  | A699 | Spirochaetal infection, unspecified |  |  |
|  |  |  |  |  |  |
|  |  |  |  |  |  |
|  |  | A97 | Dengue fever [classical dengue] | 061. | Dengue fever |
|  |  | B37 | Candidiasis |  |  |
|  |  | B40 | Blastomycosis | 116A | Blastomycosis |
|  |  | B41 | Paracoccidioidomycosis | 116B | Paracoccidioidomycosis |
|  |  | B42 | Sporotrichosis |  |  |
|  |  | B43 | Chromomycosis and chromomycotic abscess |  |  |
|  |  | B44 | Aspergillosis | 117D | Aspergillosis |
|  |  | B45 | Cryptococcosis | 117F | Cryptococcosis |
|  |  | B46 | Zygomycosis |  |  |
|  |  | B48 | Other mycoses, not elsewhere classified | 116C | Lobomycosis |
|  |  | B49 | Unspecified mycosis | 111B | Tinea nigra |
|  |  |  |  | 117X | Other and unspecified mycosis |
|  |  | B50 | Plasmodium falciparum malaria | 0.84 | Malaria |
|  |  | B51 | Plasmodium vivax malaria |  |  |
|  |  | B52 | Plasmodium malariae malaria |  |  |
|  |  | B53 | Other parasitologically confirmed malaria |  |  |
|  |  | B54 | Unspecified malaria |  |  |
|  |  | M00 | Pyogenic arthritis | 711A | Pyogenic arthritis |
|  |  |  |  | 711X | Unspecified infective arthritis |
|  |  | M01 | Direct infections of joint in infectious and parasitic diseases classified elsewhere | 711E | Arthropathy associated with other bacterial diseases |
|  |  |  |  | 711F | Arthropathy associated with other viral diseases |
|  |  |  |  | 711G | Arthropathy associated with mycoses |
|  |  |  |  | 711H | Arthropathy associated with helminthiasis |
|  |  |  |  | 711W | Arthropathy associated with other specified infectious and parasitic diseases |
|  |  | M86 | Osteomyelitis | 730A | Acute osteomyelitis |
|  |  |  |  | 730B | Chronic osteomyelitis |
|  |  |  |  | 730C | Unspecified osteomyelitis |
|  |  |  |  | 730D | Periostitis without mention of osteomyelitis |
|  |  |  |  | 730X | Unspecified infection of bone |
|  |  | T802 | Infections following infusion, transfusion and therapeutic injection | 999D | Infection, sepsis or septicaemia caused by infusion, injection, transfusion or vaccination |
|  |  | T814 | Infection following a surgical or medical procedure, not elsewhere classified | 998F | Postoperative infection |
|  |  | T880 | Infection following immunization |  |  |
|  |  | A50 | Congenital syphilis |  |  |
|  |  | A65 | Nonvenereal syphilis |  |  |
|  |  | A74 | Other diseases caused by chlamydiae | 077A | Inclusion conjunctivitis |
|  |  | A75 | Epidemic typhus | 080-081 | 080 Epidemic typhus 081 Other typhus |
|  |  | A77 | Spotted fever [tick-borne rickettsioses] | 082-083 | 082 Tick-borne rickettsioses 083 Other rickettsioses |
|  |  | A78 | Q fever |  |  |
|  |  | A79 | Other rickettsioses |  |  |
|  |  | A80 | Acute poliomyelitis | 045. | Acute poliomyelitis |
|  |  | A92 | Other mosquito-borne viral fevers except dengue | 065-066 | 065 Arthropod-borne hemorrhagic fever 066 Other arthropod-borne viral diseases |
|  |  | A93 | Other arthropod-borne viral fevers, not elsewhere classified | 088. | Other arthropod-borne diseases |
|  |  |  |  | 074W | Other specified diseases due to Coxsackie virus |
|  |  | A94 | Unspecified arthropod-borne viral fever |  |  |
|  |  | A95 | Yellow fever | 0.60 | Yellow fever |
|  |  | A96 | Arenaviral haemorrhagic fever | 078H | Arenaviral haemorrhagic fever |
|  |  |  |  | 078W | Other viral diseases |
|  |  | A98 | Other viral haemorrhagic fevers, not elsewhere classified | 078G | Hemorrhagic nephrosonephritis |
|  |  | A99 | Unspecified viral haemorrhagic fever |  |  |
|  |  | B01 | Varicella [chickenpox] | 052. | Varicella [chickenpox] |
|  |  | B02 | Zoster [herpes zoster] | 053. | Shingles / Herpes Zoster |
|  |  | B03 | Smallpox | 050. | Smallpox |
|  |  | B04 | Monkeypox |  |  |
|  |  | B05 | Measles | 055. | Measles |
|  |  | B06 | Rubella | 056. | Rubella |
|  |  | B25 | Cytomegaloviral disease | 078F | Cytomegaloviral disease |
|  |  | B26 | Mumps | 072. | Mumps |
|  |  | B27 | Infectious mononucleosis | 075. | Infectious mononucleosis |
|  |  | B33 | Other viral diseases, not elsewhere classified | 074B | Epidemic pleurodynia |
|  |  |  |  | 074C | Coxsackie carditis |
|  |  |  |  | 079W | Other specified viral infections |
|  |  |  |  | 079X | Unspecified viral infections |
|  |  |  |  | 062. | Mosquito-borne viral encephalitis |
|  |  | B34 | Viral infection of unspecified site | 079A | Adenovirus infection in conditions classified elsewhere and of unspecified site |
|  |  |  |  | 079B | Echo virus infection in conditions classified elsewhere and of unspecified site |
|  |  |  |  | 079C | Coxsackie virus infection in conditions classified elsewhere and of unspecified site |
|  |  |  |  | 079D | Rhinovirus infection in conditions classified elsewhere and of unspecified site |
|  |  |  |  | 079W | Other specified viral infections |
|  |  |  |  | 079X | Unspecified viral infections |
|  |  |  |  | 790W | Viremia, unspecified |
|  |  | B55 | Leishmaniasis | 085. | Leishmaniasis |
|  |  | B58 | Toxoplasmosis | 130 | Toxoplasmosis |
|  |  | B60 | Other protozoal diseases, not elsewhere classified | 136C | Specific infections by free-living amebae |
|  |  |  |  | 136W | Other specified infectious and parasitic diseases |
|  |  | B64 | Unspecified protozoal disease | 136X | Unspecified infectious and parasitic diseases |
|  |  | B67 | Echinococcosis | 122 | Echinococcosis |
|  |  | B95 | Streptococcus and staphylococcus as the cause of diseases classified to other chapters | 041A | Streptococcus infection in conditions classified elsewhere and of unspecified site |
|  |  |  |  | 041B | Staphylococcus infection in conditions classified elsewhere and of unspecified site |
|  |  |  |  | 041C | Pneumococcus infection in conditions classified elsewhere and of unspecified site |
|  |  | B96 | Other specified bacterial agents as the cause of diseases classified to other chapters | 041A | Streptococcus infection in conditions classified elsewhere and of unspecified site |
|  |  |  |  | 041B | Staphylococcus infection in conditions classified elsewhere and of unspecified site |
|  |  |  |  | 041C | Pneumococcus infection in conditions classified elsewhere and of unspecified site |
|  |  |  |  | 041D | Friedländer's bacillus infection in conditions classified elsewhere and of unspecified site |
|  |  |  |  | 041E | Escherichia coli [e. coli] infection in conditions classified elsewhere and of unspecified site |
|  |  |  |  | 041F | Hemophilus influenzae [H. influenzae] infection in conditions classified elsewhere and of unspecified site |
|  |  |  |  | 041G | Proteus (mirabilis) (morganii) infection in conditions classified elsewhere and of unspecified site |
|  |  |  |  | 041H | Pseudomonas infection in conditions classified elsewhere and of unspecified site |
|  |  |  |  | 041W | Other specified bacterial infections in conditions classified elsewhere and of unspecified site |
|  |  | B97 | Viral agents as the cause of diseases classified to other chapters | 079A | Adenovirus infection in conditions classified elsewhere and of unspecified site |
|  |  |  |  | 079B | Echo virus infection in conditions classified elsewhere and of unspecified site |
|  |  |  |  | 079C | Coxsackie virus infection in conditions classified elsewhere and of unspecified site |
|  |  |  |  | 079D | Rhinovirus infection in conditions classified elsewhere and of unspecified site |
|  |  |  |  | 079W | Other specified viral infections |
|  |  |  |  | 079X | Unspecified viral infections |
|  |  | B98 | Other specified infectious agents as the cause of diseases classified to other chapters | | |
|  |  | B99 | Other and unspecified infectious diseases |  |  |
|  |  | P35 | Congenital viral diseases | 771A | Congenital rubella |
|  |  |  |  | 771B | Congenital cytomegalovirus infection |
|  |  |  |  | 771C | Other congenital infections specific to the perinatal period |
|  |  | P37 | Other congenital infectious and parasitic diseases | 771H | Neonatal Candida infection |
|  |  | P39 | Other infections specific to the perinatal period | 771F | Neonatal infective mastitis |
|  |  |  |  | 771G | Neonatal conjunctivitis and dacryocystitis |
|  | **Pregnancy-associated** | O753 | Other infection during labour | 659D | Generalized infection during labor |
|  |  | O85 | Puerperal fever | 670 | Major puerperal infection |
|  |  | O030 | Spontaneous abortion; Incomplete, complicated by genital tract and pelvic infection | | |
|  |  | O035 | Spontaneous abortion; Complete or unspecified, complicated by genital tract and pelvic infection | | |
|  |  | O040 | Legal abortion; Incomplete, complicated by genital tract and pelvic infection | |  |
|  |  | O045 | Legal abortion; Complete or unspecified, complicated by genital tract and pelvic infection | | |
|  |  | O050 | Other abortion; Incomplete, complicated by genital tract and pelvic infection | |  |
|  |  | O055 | Other abortion; Complete or unspecified, complicated by genital tract and pelvic infection | | |
|  |  | O060 | unspecified abortion; Incomplete, complicated by genital tract and pelvic infection | | |
|  |  | O065 | Unspecified abortion; Complete or unspecified, complicated by genital tract and pelvic infection | | |
|  |  | O070 | Failed medical abortion, complicated by genital tract and pelvic infection | |  |
|  |  | O075 | Other and unspecified failed attempted abortion, complicated by genital tract and pelvic infection | | |
|  |  | O080 | Genital tract and pelvic infection following abortion and ectopic and molar pregnancy | | |
|  |  | O86 | Other puerperal infections |  |  |
|  |  | O23 | Infections of genitourinary tract in pregnancy | 646G | Infections of genitourinary tract in pregnancy |
|  |  | O411 | Infection of amniotic sac and membranes | 658E | Infection of amniotic cavity, membranes and placenta |
|  |  | O883 | Obstetric septic embolism | 673D | Obstetric septic embolism |
|  |  | O91 | Infections of breast associated with childbirth | 675A | Infections of nipple associated with childbirth |
|  |  |  |  | 675B | Abscess of breast associated with childbirth |
|  |  |  |  | 675C | Superficial mastitis associated with childbirth |
|  |  | O98 | Maternal infectious and parasitic diseases classifiable elsewhere but complicating pregnancy, childbirth and the puerperium | 016E | Tuberculous oophoritis and salpingitis |
|  |  |  |  | 095. | Other forms of late syphilis with symptoms |
|  |  |  |  | 647B | Gonorrhea complicating pregnancy childbirth or the puerperium |
|  |  |  |  | 647C | Other venereal diseases complicating pregnancy childbirth or the puerperium |
|  |  |  |  | 647D | Tuberculosis complicating pregnancy childbirth or the puerperium |
|  |  |  |  | 647E | Malaria complicating pregnancy childbirth or the puerperium |
|  |  |  |  | 647F | Rubella complicating pregnancy childbirth or the puerperium |
|  |  |  |  | 647G | Other viral diseases complicating pregnancy childbirth or the puerperium |
|  |  |  |  | 647W | Other specified infectious and parasitic diseases complicating pregnancy childbirth or the puerperium |
|  |  |  |  | 647X | Unspecified infection or infestation complicating pregnancy childbirth or the puerperium |
|  |  |  |  |  |  |
| **Organ dysfunction** | **Cardiovascular** | I959 | Hypotension, unspecified | 458X | Hypotension, unspecified |
|  |  | R578 | Other shock | 785F | Shock without mention of trauma |
|  |  | R579 | Shock, unspecified | 785F | Shock without mention of trauma |
|  |  |  |  |  |  |
|  | **Respiratory** | J96 | Respiratory failure, not elsewhere classified | 799A | Asphyxia and hypoxemia |
|  |  | J80 | Adult respiratory distress syndrome |  |  |
|  |  | J984 | Other disorders of lung | 518W | Other diseases of lung |
|  |  |  |  | 519B | Other diseases of trachea and bronchus not elsewhere classified |
|  |  | R060 | Dyspnoea | 786A | Dyspnea and respiratory abnormalities |
|  |  | R068 | Other and unspecified abnormalities of breathing | 786A | Dyspnea and respiratory abnormalities |
|  |  |  |  |  |  |
|  | **CNS** | F05 | Delirium, not induced by alcohol and other psychoactive substances | 293A | Acute delirium |
|  |  |  |  | 293B | Subacute delirium |
|  |  |  |  | 293W | Other specified transient organic psychosis |
|  |  |  |  | 293X | Unspecified transient organic psychosis |
|  |  |  |  | 780A | Coma and stupor |
|  |  | G931 | Anoxic brain damage, not elsewhere classified | 348B | Anoxic brain damage |
|  |  | G934 | Encephalopathy, unspecified | 323X | Unspecified causes of encephalitis, myelitis, and encephalomyelitis |
|  |  |  |  | 348D | Encephalopathy, unspecified |
|  |  | R40 | Somnolence, stupor and coma | 780A | Coma and stupor |
|  |  |  |  |  |  |
|  | **Renal** | N17 | Acute renal failure | 584F | Acute kidney failure with lesion of tubular necrosis |
|  |  |  |  | 584G | Acute kidney failure with lesion of renal cortical necrosis |
|  |  |  |  | 584H | Acute kidney failure with lesion of renal medullary [papillary] necrosis |
|  |  |  |  | 584W | Acute kidney failure with other specified pathological lesion in kidney |
|  |  |  |  | 584X | Acute kidney failure, unspecified |
|  |  | N19 | Unspecified kidney failure | 586 | Unspecified kidney failure |
|  | **Metabolic** | E872 | Acidosis | 276C | Acidosis |
|  | **Hematological** | D65 | Disseminated intravascular coagulation [defibrination syndrome] | 286G | Defibrineringssyndrom |
|  |  | D688 | Other specified coagulation defects | 286X | Other and unspecified coagulation defects |
|  |  | D689 | Coagulation defect, unspecified | 286X | Other and unspecified coagulation defects |
|  |  | D695 | Secondary thrombocytopenia | 287E | Secondary thrombocytopenia |
|  |  | D696 | Thrombocytopenia, unspecified | 287F | Thrombocytopenia, unspecified |
|  |  | K720 | Acute and subacute hepatic failure | 570 | Acute and subacute necrosis of liver |
|  |  |  |  |  |  |
|  | **Hepatic** | K729 | Hepatic failure, unspecified | 570 | Acute and subacute necrosis of liver |
|  |  | K762 | Central haemorrhagic necrosis of liver | 570 | Acute and subacute necrosis of liver |
|  |  | K763 | Infarction of liver | 573E | Infarction of liver |
|  |  |  |  |  |  |
|  | **Organ dysfunction neonates** | P60 | Disseminated intravascular coagulation of fetus and newborn | 776C | Disseminated intravascular coagulation in newborn |
|  |  | P616 | Other transient neonatal disorders of coagulation | 776D | Other transient neonatal disorders of coagulation |
|  |  | P610 | Transient neonatal thrombocytopenia | 776B | Transient neonatal thrombocytopenia |
|  |  | P612 | Anaemia of prematurity | 776G | Anaemia of prematurity |
|  |  | P544 | Neonatal adrenal haemorrhage | 772F | Adrenal haemorrhage of fetus or newborn |
|  |  | P58.2 | Neonatal jaundice due to infection | 774B | Perinatal jaundice from other excessive hemolysis |
|  |  |  |  |  |  |
|  |  | P592 | Neonatal jaundice from other and unspecified causes | 774E | Perinatal jaundice due to hepatocellular damage |
|  |  | P598 | Other specified intestinal obstruction of newborn | 774E | Perinatal jaundice due to hepatocellular damage |
|  |  |  |  |  |  |
|  |  | P768 | Perinatal intestinal perforation |  |  |
|  |  | P780 | Perinatal intestinal perforation | 777G | Perinatal intestinal perforation |
|  |  | P52 | Intracranial nontraumatic haemorrhage of fetus and newborn | 772B | Intraventricular hemorrhage of fetus or newborn |
|  |  |  |  | 772C | Subarachnoid hemorrhage of fetus or newborn |
|  |  |  |  | 767A | Subdural and cerebral hemorrhagem, birth trauma |
|  |  | P25 | Interstitial emphysema and related conditions originating in the perinatal period | 770C | Interstitial emphysema and related conditions originating in the perinatal period |
|  |  |  |  | 770F | Other and unspecified atelectasis of fetus and newborn |
|  |  | P26 | Pulmonary haemorrhage originating in the perinatal period | 770D | Pulmonary hemorrhage of fetus and newborn |
|  |  | P281 | Other and unspecified atelectasis of newborn | 770F | Other and unspecified atelectasis of fetus and newborn |
|  |  | P282 | Cyanotic attacks of newborn | 770W | Other respiratory problems after birth |
|  |  | P285 | Respiratory failure of newborn | 770W | Other respiratory problems after birth |
|  |  | P288 | Other specified respiratory conditions of newborn | 770W | Other respiratory problems after birth |
|  |  | P290 | Neonatal cardiac failure |  |  |
|  |  | P293 | Persistent fetal circulation | 747A | Patent ductus arteriosus |
|  |  | P294 | Transient myocardial ischaemia of newborn |  |  |
|  |  | P298 | Other cardiovascular disorders originating in the perinatal period | |  |
|  |  | P299 | Cardiovascular disorder originating in the perinatal period, unspecified | |  |
|  |  | P704 | Other neonatal hypoglycaemia | 775G | Neonatal hypoglycemia |
|  |  | P740 | Late metabolic acidosis of newborn | 775H | Late metabolic acidosis of newborn |
|  |  | P742 | Disturbances of sodium balance of newborn | 775F | Other transitory neonatal electrolyte disturbances |
|  |  | P808 | Other hypothermia of newborn |  |  |
|  |  | P809 | Unspecified hypothermia of newborn |  |  |
|  |  | P819 | Disturbance of temperature regulation of newborn, unspecified | 778E | Other disturbances of temperature regulation of newborn |
|  |  | P90 | Convulsions of newborn | 779A | Convulsions of newborn |
|  |  | P91 | Other disturbances of cerebral status of newborn | 779B | Other and unspecified cerebral irritability in newborn |
|  |  |  |  | 779C | Cerebral depression, coma, and other abnormal cerebral signs in fetus or newborn |

## eTable 2, Table of ICD-codes in ICD-10-SE and ICD-9 SE Used for the Outcome Analysis and Grouped by Chapter

| **Chapters of ICD-10-SE with a selection of codes for each chapter and additional codes for associated symtoms (R-codes) and laboratory findings** | **ICD-10-SE-codes** | **Corresponding codes in ICD-9 SE** |
| --- | --- | --- |
| **1. Infektionssjukdomar (Infectious diseases), including indectious diseases from other chapters** |  |  |
| INFEKTIONSSJUKDOMAR UTGÅENDE FRÅN MAG-TARMKANALEN (Intestinal infectious diseases) | A00-A09 | 001-009 Infektionssjukdomar och parasitsjukdomar: 001-009 |
| TUBERKULOS (Tuberculosis) | A15-A19 | 010-018 Tuberkulos: 010-018 |
| ANDRA BAKTERIESJUKDOMAR (Other bacterial diseases) | A30-A49 | 030-033, 034B, 035-041 Andra bakteriesjukdomar 030-033, 034B, 035-041 |
| VIRUSSJUKDOMAR I CENTRALA NERVSYSTEMET (Viral infections of the central nervous system ) | A80-A89 | 045-049, 62-64 071 045 Polio 046 Slow virus-infektion i CNS,  047 Meningit orsakad av enterovirus 048 Andra sjukdomar i centrala nervsystemet orsakade av enterovirus 049 Andra virussjukdomar i centrala nervsystemet 062 Virusencefalit överförd av myggor 063 Virusencefalit överförd av fästingar 064 Virusencefalit överförd av andra och ospecificerade leddjur 071 Rabies |
| VIRUSSJUKDOMAR MED HUDUTSLAG OCH SLEMHINNEUTSLAG (Viral infections characterized by skin and mucous membrane lesions) | B00-B09 | 050-057, 074A, 074D, 078B 050-057 Virussjukdomar med hudutslag  074A Herpangina 074D Hand, foot and mouth disease 078B Virusvårtor |
| VIRUSHEPATIT (Viral hepatitis) | B15-B19 | 070 070 Virushepatit |
| SJUKDOM ORSAKAD AV HUMANT IMMUNBRISTVIRUS (Human immunodeficiency virus [HIV] disease) | B20-B24 | 079J, 279K 279K Acquired immune deficiency syndrome (AIDS) 079J (Sjukdom orsakat av HTLV-III virus, disease caused by the HTLV-III virus) |
| ANDRA VIRUSSJUKDOMAR (Other viral diseases) | B25-B34 | 072, 074B, 074C, 074W, 075, 077, 078F, 079 072 Påssjuka 074B Epidemisk pleurodyni Bornholmssjuka Epidemisk myalgi Epidemisk myosit 074C Coxsackiekardit 074W Andra specifika sjukdomar orsakade av Coxsackievirus 075 Körtelfeber 077 Andra sjukdomar i ögats bindhinna orsakade av virus och klamydier 078F Cytomegalvirussjukdom 079 Virusinfektioner vid sjukdomar som klassificeras annorstädes och med ospecificerad lokalisation |
| SENA EFFEKTER AV INFEKTIONSSJUKDOMAR OCH PARASITSJUKDOMAR (Sequelae of infectious and parasitic diseases) | B90-B94 | 137-139  Sena effekter av infektionssjukdomar och parasitsjukdomar |
| BAKTERIER, VIRUS OCH ANDRA INFEKTIÖSA ORGANISMER (Bacterial, viral and other infectious agents) | B95-B98 | 041, 079 041 Bakterieinfektion vid sjukdomstillstånd som klassificeras T annorstädes och med icke specificerad lokalisation 079 Virusinfektioner vid sjukdomar som klassificeras annorstädes och med ospecificerad lokalisation |
| ANDRA INFEKTIONSSJUKDOMAR (Other infectious diseases) | B99 | 136W, 136X 136W Annan specificerad infektionssjukdom eller parasitsjukdom Candiruinfektion 136X Infektionssjukdom eller parasitsjukdom, ospecificerad |
| AKUTA INFEKTIONER I ÖVRE LUFTVÄGARNA (Acute upper respiratory infections) | J00-J06 | 460-465, 034A 034A Streptokocktonsillit 460 Akut nasofaryngit 461 Akut sinuit 462 Akut faryngit 463 Akut tonsillit 464 Akut laryngit 465 Akut övre luftvägsinfektion, multipel eller ospecificerad lokalisation |
| Nedre luftvägsinfektioner (Lower respiratory tract infections) | J09-J18, J20-J22, J85-J86 | 466, 480-487, 510, 511B, 513 466 Akut luftrörskatarr Bronchitis et bronchiolitis acuta 480-487 Lunginflammation och influensa 510 Empyem 511B Pleurit med utgjutning med uppgift om annan bakteriell orsak än T tuberkulos 513 Abscess i lunga och mediastinum |
| Hudinfektioner (Skin infections) | L00-L08 | 680-686 Infektioner i hud och underhud |
| Ledinfektioner (Joint infections) | M00-M03 | 711A, 711B 711D, 711E, 711F, 711G, 711H, 711W, 711X 711A Varig artrit 711B Uroartropati |
|  |  |  |
| **2. Tumörer (Neoplasms)** |  |  |
| Maligna tumörer i läpp, munhåla och svalg (Malignant neoplasms of lip, oral cavity and pharynx ) | C00-C14 | 140-149 |
|  |  |  |
| Malign tumör i matsmältningsorganen (Malignant neoplasms of digestive organs) | C15-C26 | 150-159 |
| Malign tumör i andningsorganen och brösthålans organ (Malignant neoplasms of respiratory and intrathoracic organs) | C30-C39 | 160-165 Malign tumör i andningsorganen och brösthålans organ |
| Maligna tumörer i ben och ledbrosk (Malignant neoplasms of bone and articular cartilage) | C40-C41 | 170 Malign tumör i ben och ledbrosk |
| Melanom och andra maligna tumörer i huden (Melanoma and other malignant neoplasms of skin) | C43-C44 | 172-173 172 Malignt melanom (pigmentsvulst) i huden 173 Andra maligna tumörer i huden |
| Maligna tumörer i mesotelial vävnad och mjukvävnad (Malignant neoplasms of mesothelial and soft tissue) | C45-C49 | 171 Malign tumör i bindväv och annan mjukvävnad |
| Malign tumör i bröstkörtel (Malignant neoplasm of breast) | C50 | 174-175 174 Malign tumör i kvinnlig bröstkörtel 175 Malign tumör i manlig bröstkörtel |
| Maligna tumörer i de kvinnliga könsorganen (Malignant neoplasms of female genital organs) | C51-C58 | 179-184 |
| Maligna tumörer i de manliga könsorganen (Malignant neoplasms of male genital organs) | C60-C63 | 185-187 |
| Maligna tumörer i urinorganen (Malignant neoplasms of urinary tract) | C64-C68 | 188-189 |
| Maligna tumörer i öga, hjärnan och andra delar av centrala nervsystemet (Malignant neoplasms of eye, brain and other parts of central nervous system) | C69-C72 | 190-192 190 Malign tumör i öga 191 Malign tumör i hjärnan 192 Malign tumör i andra och icke specificerade delar av nervsystemet |
| Maligna tumörer i tyreoidea och andra endokrina körtlar (Malignant neoplasms of thyroid and other endocrine glands) | C73-C75 | 193-194 193 Malign tumör i tyreoidea 194 Malign tumör i andra endokrina körtlar och därmed besläktade vävnader |
| Maligna tumörer med ofullständigt angivna, sekundära och ospecificerade lokalisationer (Malignant neoplasms of ill-defined, secondary and unspecified sites) | C76-C80 | 195-199 195 Malign tumör med annan och ofullständigt angiven lokalisation 196 Sekundär och icke specificerad malign tumör i lymfkörtlarna 197 Sekundär malign tumör i andningsorganen och T matsmältningsorganen 198 Sekundär malign tumör med andra specificerade lokalisationer 199 Malign tumör utan specificerad lokalisation |
| Maligna tumörer i lymfatisk, blodbildande och besläktad vävnad (Malignant neoplasms, stated or presumed to be primary, of lymphoid, haematopoietic and related tissue) | C81-C96 | 200-208 Malign tumör i lymfatisk och blodbildande vävnad |
| Flera (primära) maligna tumörer med olika ursprung (Malignant neoplasms of independent (primary) multiple sites) | C97 | Finns ej motsvarighet i ICD9 |
| Cancer in situ (In situ neoplasms) | D00-D09 | 230-234 230-234 Cancer in situ (lokalt begränsad cancer |
| Benigna tumörer (Benign neoplasms) | D10-D36 | 210-229 210-229 Benigna (godartade) tumörer |
| Tumörer av osäker eller okänd natur (Neoplasms of uncertain or unknown behaviour) | D37-D48 | 235 -239 235-238 Tumörer av oklar natur 239 Tumör av icke specificerad natur |
|  |  |  |
| **3. Sjukdomar i blod och blodbildande organ (Diseases of the blood and blood-forming organs)** |  |  |
| Nutritionsanemi (Nutritional anaemias) | D50-D53 | 280-281 280 Järnbristanemi 281 Andra bristanemier |
| Hemolytiska anemier (Hemolytic anemias) | D55-D59 | 282-283 282 Ärftliga hemolytiska anemier 283 Förvärvade hemolytiska anemier |
| Aplastisk anemi och andra anemier (Aplastic and other anaemias) | D60-D64 | 284-285 284 Aplastisk anemi 285 Andra och ospecificerade anemier |
| Koagulationsrubbningar, purpura och andra blödningstillstånd (Coagulation defects, purpura and other haemorrhagic conditions) | D65-D69 | 286-287 286 Koagulationsrubbningar 287 Purpura |
| Andra sjukdomar i blod och blodbildande organ (Other diseases of blood and blood-forming organs) | D70-D77 | 288-289 288 Sjukdomar i vita blodkroppar 289 Andra sjukdomar i blod och blodbildande organ |
| Vissa rubbningar i immunsystemet (Certain disorders involving the immune mechanism) | D80-D89 | 135, 273A, 273B, 273C, 279 135 Sarkoidos 273A Polyklonal hypergammaglobulinemi 273B Monoklonal paraproteinemi 273C Andra paraproteinemier 279 Immunbristsjukdomar |
| Onormala fynd vid blodundersökning utan diagnos (Abnormal findings on examination of blood, without diagnosis) | R70-R79 | 790A, 790B, 790C, 790D, 790E, 790F, 790G, 790X, 796A 790A Onormala röda blodkroppar 790B Förhöjd sänkningsreaktion 790C Onormalt glykostoleranstest 790D Förhöjd alkoholnivå i blodet 790E Ospecifik förhöjning av transaminaser, mjölksyredehydrogenas och kreatinkinas i blodet 790F Annan icke specificerad onormal enzymnivå i serum 790G Annan onormal blodkemi 790X Annat icke specificerat fynd vid blodundersökning 796A Ospecificerade onormala toxikologiska fynd |
|  |  |  |
| **4. Endokrina sjukdomar, nutritionsrubbningar och ämnesomsättningssjukdomar (Endocrine, nutritional and metabolic diseases)** |  |  |
| Sjukdomar i sköldkörteln (Disorders of thyroid gland ) | E00-E07 | 240-246 Sjukdomar i sköldkörteln (240-246) |
| Diabetes (Diabetes mellitus) | E10-E14 | 250 250 Diabetes |
| Andra rubbningar i glykosreglering och bukspottkörtelns inre sekretion (Other disorders of glucose regulation and pancreatic internal secretion) | E15-E16 | 251A, 251E, 251F, 251W, 251X 251A Hypoglykemiskt koma 251E Överproduktion av glukagon 251F Överproduktion av gastrin 251W Andra rubbningar i pankreas inre sekretion 251X Andra rubbningar i pankreas inre sekretion, ospecificerade |
| Sjukdomar i andra endokrina körtlar (Disorders of other endocrine glands) | E20-E35 | 252-259 252 Sjukdomar i bisköldkörtlarna 253 Rubbningar i hypofysen 254 Sjukdomar i tymus 255 Sjukdomar i binjurarna 256 Rubbningar i äggstockarnas funktion 257 Rubbningar i testiklarnas funktion 258 Samtidig rubbning i flera inresekretoriska organ 259 Andra endokrina rubbningar |
| Ämnesomsättningssjukdomar (Metabolic disorders) | E70-E90 | 270-272, 273W, 275-277 270 Rubbningar av transport och omsättning av aminosyror 271 Rubbningar i transport och omsättning av kolhydrater 272 Rubbningar i fettomsättningen 273W Andra specificerade rubbningar i plasmaproteinomsättningen 275 Rubbningar i mineralomsättningen 276 Rubbningar i vätske-, elektrolyt- och syra-basbalansen 277 Andra och ospecificerade rubbningar i ämnesomsättningen |
|  |  |  |
| **5. Psykiska sjukdomar och syndrom samt beteendestörningar (Mental and behavioural disorders)** | |  |
| Organiska, inklusive symtomatiska, psykiska störningar (Organic, including symptomatic, mental disorders) | F00-F09 | 290, 293, 294, 310 290 Demens i samband med åldrandet 293 Övergående organiska psykoser 294 Andra organiska psykoser (kroniska) |
| Psykiska störningar och beteendestörningar orsakade av psykoaktiva substanser (Mental and behavioural disorders due to psychoactive substance use) | F10-F19 | 291-292, 303-305 291 Alkoholpsykoser 292 Drogpsykoser 303 Alkoholberoende 304 Drogberoende 305 Missbruk av alkohol, droger och läkemedel utan beroende |
| Schizofreni, schizotypa störningar och vanföreställningssyndrom (Schizophrenia, schizotypal and delusional disorders) | F20-F29 | 295, 297-298 295 Schizofreni 297 Paranoida tillstånd 298 Reaktiva psykoser |
| Förstämningssyndrom (Mood [affective] disorders) | F30-F39 | 296, 311 296 Affektiv psykos 311 Depressiva störningar som ej klassificeras annorstädes |
| Neurotiska, stressrelaterade och somatoforma syndrom (Neurotic, stress-related and somatoform disorders) | F40-F48 | 300, 306, 308-309 300 Neuroser 306 Psykofysiologisk reaktion 308 Katastrofreaktion 309 Krisreaktion |
| Beteendestörningar förenade med fysiologiska rubbningar och fysiska faktorer (Behavioural syndromes associated with physiological disturbances and physical factors) | F50-F59 | 307B, 307F, 307E 302 Sexuella störningar 307B Anorexia nervosa 307F Andra och ospecificerade ätproblem 307E Specifika sömnstörningar |
| Personlighetsstörningar och beteendestörningar hos vuxna (Disorders of adult personality and behaviour) | F60-F69 | 301-302 301 Personlighetsstörningar 302 Sexuella störningar |
| Psykisk utvecklingsstörning (Mental retardation) | F70-F79 | 317-319 Psykisk utvecklingsstörning (317-319) |
| Störning i psykisk utveckling (Disorders of psychological development) | F80-F89 | 299, 315 299 Barndomspsykoser 315 Specifika störningar i utvecklingen |
| Beteendestörningar och emotionella störningar med debut vanligen under barndom och ungdomstid (Behavioural and emotional disorders with onset usually occurring in childhood and adolescence) | F90-F98 | 307A, 307C, 307D, 307G, 307H, 312-314 307A Stamning 307C Tics 307D Rörelsestereotypier 307G Enures 307H Enkopres 312 Uppföranderubbning som ej klassificeras annorstädes 313 Emotionella störningar specifika för barndom och ungdomsår 314 Hyperkinetiska syndrom i barndomen |
| Ospecificerad psykisk störning (Unspecified mental disorder) | F99 | 307X 307X Andra och ospecificerade psykiska symtom och syndrom |
| Symtom och sjukdomstecken avseende intellektuella funktioner, uppfattningsförmåga, känsloläge och beteende (Symptoms and signs involving cognition, perception, emotional state and behaviour) | R40-R46 | 780A, 780B 780A Koma och stupor 780B Hallucinationer 780E Yrsel och svindel 780F Sömnstörningar |
|  |  |  |
| **6. Sjukdomar i nervsystemet (Diseases of the nervous system)** |  |  |
| Inflammatoriska sjukdomar i centrala nervsystemet (Inflammatory diseases of the central nervous system) | G00-G09 | 320-326 Inflammatoriska sjukdomar i centrala nervsystemet (320-326) |
| Systemiska atrofier som primärt engagerar centrala nervsystemet (Systemic atrophies primarily affecting the central nervous system) | G10-G14 | 334-335 334 Sjukdom i både ryggmärg och lillhjärna 335 Motorneuronsjukdomar |
| Basalgangliesjukdomar och rörelserubbningar (Extrapyramidal and movement disorders) | G20-G26 | 332-333 332 Parkinsons sjukdom 333 Annan extrapyramidal sjukdom och rörelserubbning |
| Andra degenerativa sjukdomar i nervsystemet (Other degenerative diseases of the nervous system) | G30-G32 | 330W, 330X, 331A, 331B, 331C, 331W, 331X 330W Andra cerebrala degenerationer manifesterade i barndomen 330X Cerebral degeneration manifesterad i barndomen, ospecificerad 331A Presenil och senil degeneration av Alzheimer-typ 331B Picks sjukdom 331C Senil degeneration av hjärnan av ospecificerad typ 331W Annan specificerad cerebral degeneration 331X Cerebral degeneration, ospecificerad |
| Myelinförstörande sjukdomar i centrala nervsystemet (Demyelinating diseases of the central nervous system) | G35-G37 | 340-341 340 Multipel skleros 341 Andra myelinförstörande sjukdomar i centrala nervsystemet |
| Episodiska och paroxysmala sjukdomar (Episodic and paroxysmal disorders) | G40-G47 | 345-347, 435 345 Epilepsi 346 Migrän och annan vaskulär huvudvärk 347 Kataplexi och narkolepsi 435 Övergående cerebral ischemi |
| Sjukdomar i nerver, nervrötter och nervplexus (Nerve, nerve root and plexus disorders) | G50-G59 | 350-355 350 Sjukdomar i trigeminusnerven 351 Sjukdomar i facialisnerven 352 Sjukdomar i andra kranialnerver 353 Sjukdomar i nervrötter och nervplexus 354 Mononeuropati 355 Mononeuropati |
| Polyneuropatier och andra sjukdomar i perifera nervsystemet (Polyneuropathies and other disorders of the peripheral nervous system) | G60-G64 | 356-357 356 rftlig och idiopatisk perifer neuropati 357 Inflammatorisk och toxisk neuropati |
| Neuromuskulära transmissionsrubbningar och sjukdomar i muskler (Diseases of myoneural junction and muscle) | G70-G73 | 358-359 358 Myoneurala sjukdomar 359 Muskeldystrofi och andra muskelsjukdomar |
| Cerebral pares och andra förlamningssyndrom (Cerebral palsy and other paralytic syndrome) | G80-G83 | 342-344 342 Ensidig förlamning 343 Cerebral pares 344 Andra förlamningssyndrom |
| Andra sjukdomar i nervsystemet (Other disorders of the nervous system) | G90-G99 | 331D Kommunicerande hydrocefalus Utesluter: Medfödd hydrocefalus (741A, 742D) 331E Obstruktiv hydrocefalus 336 Andra sjukdomar i ryggmärgen 337 Sjukdomar i autonoma nervsystemet 348 Andra sjukdomstillstånd i hjärnan 349 Andra och icke specificerade sjukdomar i nervsystemet |
| Symtom och sjukdomstecken från nervsystemet och muskuloskeletala systemet (Symptoms and signs involving the nervous and musculoskeletal systems) | R25-R29 | 781A, 781C, 781D, 781E, 781G, 781H, 781X 781A Abnorma ofrivilliga rörelser 781C Gångrubbningar 781D Koordinationsrubbning 781E @vergående förlamning i extremitet 781G Meningism 781H Tetani 781X Andra symtom från nervsystemet och muskuloskeletala systemet |
|  |  |  |
| **7. Sjukdomar i ögat och närliggande organ (Diseases of the eye and adnexa)** | H00-H59 | 360-379 Sjukdomar i ögat och närliggande organ (360-379) |
|  |  |  |
| **8. Sjukdomar i örat och mastoidutskottet (Diseases of the ear and mastoid process)** | H60-H95 | 380-389 Sjukdomar i örat och mastoidutskottet (380-389) |
| (Ytterörat och hörselgång |  |  |
| Mellanöra och mastoid |  |  |
| Innerörat |  |  |
| Övriga) |  |  |
|  |  |  |
| **9. Cirkulationsorganens sjukdomar (Diseases of the circulatory system)** | |  |
| Akut reumatisk feber & Kroniska reumatiska hjärtsjukdomar (Acute rheumatic fever & Chronic rheumatic heart diseases) | I00-I09 | 390-398 Akut reumatisk feber (390-392) Kroniska reumatiska hjärtsjukdomar (393-398) |
| Hypertonisjukdomar (Hypertensive diseases) | I10-I15 | 401-405 Hypertonisjukdomar (högt blodtryck och därmed sammanhängande sjukdomar) (401-405) |
| Ischemiska hjärtsjukdomar (Ischaemic heart diseases) | I20-I25 | 410-414 Ischemisk hjärtsjukdom (sjukdom orsakad av otillräcklig blodtillförsel till hjärtmuskeln) (410-414) |
| Sjukdomstillstånd inom lungcirkulationen (Pulmonary heart disease and diseases of pulmonary circulation) | I26-I28 | 415-417 Sjukdomstillstånd inom lungcirkulationen (415-417) |
| Andra former av hjärtsjukdom (Other forms of heart disease) | I30-I52 | 420-429 Andra hjärtsjukdomar (420-429) |
| Sjukdomar i hjärnans kärl (Cerebrovascular diseases) | I60-I69 | 430-434, 436-438 Sjukdomar i hjärnans kärl (430-438) (Ej 435, ligger under CNS) 436 Akut ofullständigt preciserad cerebrovaskulär sjukdom |
| Sjukdomar i artärer, arterioler och kapillärer (Diseases of arteries, arterioles and capillaries) | I70-I79 | 440-445, 447-448 Sjukdomar i artärer, arterioler (småartärer) och kapillärer (hårrörskärl) (440-448) |
| Sjukdomar i vener, lymfkärl och lymfkörtlar som ej klassificeras på annan plats (Diseases of veins, lymphatic vessels and lymph nodes, not elsewhere classified) | I80-I89 | 451-457 Sjukdomar i vener och lymfkärl samt andra sjukdomar i cirkulationsorganen (451-459) |
| Andra och ospecificerade sjukdomar i cirkulationsorganen (Other and unspecified disorders of the circulatory system) | I95-I99 | 458-459 458 Hypotoni (lågt blodtryck) 459 Andra sjukdomar i cirkulationsorganen |
| Symtom och sjukdomstecken från cirkulationsorganen och andningsorganen (Symptoms and signs involving the circulatory and respiratory systems) | R00-R09 | 785A, 785B, 785C, 785D, 785E, 786  785A Icke specificerad takykardi 785B Hjärtklappning 785C Funktionella och icke specificerade hjärtblåsljud 785D Andra onormala hjärtljud 785E Gangrän 786 Symtom från andningsorganen och andra bröstorgan |
|  |  |  |
| **10. Andningsorganens sjukdomar (Diseases of the respiratory system)** | |  |
| Andra sjukdomar i övre luftvägarna (Other diseases of upper respiratory tract) | J30-J39 | 470-478 Andra sjukdomar i övre luftvägarna (470-478) |
| Kroniska sjukdomar i nedre luftvägarna (Chronic lower respiratory diseases) | J40-J47 | 490-494, 496 Kronisk obstruktiv lungsjukdom och därmed sammanhängande tillstånd (490-496) (EJ 495 Exogen allergisk alveolit, ligger under J67 i ICD10) |
| Andra lungsjukdomar som huvudsakligen engagerar interstitiet (Other respiratory diseases principally affecting the interstitium) | J80-J84 | 515-516, 518D 515 Bindvävsomvandling av lungorna efter inflammation 516 Annan alveolär lungsjukdom 518D Lungeosinofili |
| Andra sjukdomar i lungsäcken (Other diseases of pleura) | J90-J94 | 511A, 511J, 511K, 511W, 511X, 512 511A Pleurit utan uppgift om vätskeutgjutning eller aktuell tuberkulos 511J Pleuraplack 511K Pleurainrullning 511W Annan specificerad pleurautgjutning, utom tuberkulos 511X Utgjutning i lungsäcken, ospecificerad 512 Pneumotorax |
| Andra sjukdomar i andningsorganen (Other diseases of the respiratory system) | J95-J99 | 514, 517-519 514 Blodöverfyllnad i lungorna 517 Lungengagemang vid sjukdomar som klassificeras annorstädes 518 Andra lungsjukdomar 519 Andra sjukdomar i andningsorganen |
|  |  |  |
| **11. Matsmältningsorganens sjukdomar (Diseases of the digestive system)** | |  |
| Sjukdomar i munhåla, spottkörtlar och käkar (Diseases of oral cavity, salivary glands and jaws) | K00-K14 | 520-529 Sjukdomar i munhåla, spottkörtlar och käkar (520-529) |
| Matstrupens, magsäckens och tolvfingertarmens sjukdomar (Diseases of oesophagus, stomach and duodenum) | K20-K31 | 530-537 Matstrupens, magsäckens och tolvfingertarmens sjukdomar (530-537) |
| Sjukdomar i blindtarmen (Diseases of appendix) | K35-K38 | 540-543 Blindtarmsinflammation (540-543) |
| Icke infektiös inflammation i tunntarmen och tjocktarmen & Andra sjukdomar i tarmen (Noninfective enteritis and colitis & Other diseases of intestines) | K50-K64 | 555-558, 560-566, 569 Icke infektiös inflammation i tunntarmen och tjocktarmen (555-558) Andra sjukdomar i tarmen och bukhinnan (560-569) |
| Sjukdomar i bukhinnan (Diseases of peritoneum) | K65-K67 | 567-568 567 Peritonit (bukhinneinflammation) 568 Andra sjukdomar i bukhinnan |
| Sjukdomar i levern (Diseases of liver) | K70-K77 | 570-573 570 Akut och subakut nekros (vävnadssönderfall) i levern 571 Kronisk leversjukdom och levercirros 572 Leverabscess och följdtillstånd vid kronisk leversjukdom 573 Andra leversjukdomar |
| Sjukdomar i gallblåsan, gallvägarna och bukspottkörteln (Disorders of gallbladder, biliary tract and pancreas) | K80-K87 | 574-577 574 Gallstenssjukdom 575 Andra sjukdomar i gallblåsan 576 Annan sjukdom i gallvägarna 577 Sjukdomar i bukspottkörteln |
| Andra sjukdomar i matsmältningsorganen (Other diseases of the digestive system) | K90-K93 | 578-579 578 Blödning i mag-tarmkanalen 579 Malabsorption |
| Symtom och sjukdomstecken från matsmältningsorganen och buken (Symptoms and signs involving the digestive system and abdomen) | R10-R19 | 782E, 787, 789 782E Ospecificerad gulsot 787 Symtom från matsmältningsorganen 789 Andra symtom från buken och bäckenet |
|  |  |  |
| **12. Hudens och underhudens sjukdomar (Diseases of the skin and subcutaneous tissue)** | |  |
| Blåsdermatoser (Bullous disorders) | L10-L14 | 694 Blåsdermatoser |
| Dermatit och eksem (Dermatitis and eczema) | L20-L30 | 690, 691, 692A, 692B, 692C, 692D, 692E, 692F, 692G, 692X, 693, 698 Andra inflammatoriska tillstånd i hud och underhud (690-698) 690 Erytematoskvamös dermatos 691 Atopisk dermatit 692 A-G & X, Kontakteksem och andra kontaktdermatiter 693 Dermatit orsakad av förtärda ämnen 698 Klåda och besläktade tillstånd |
| Papuloskvamösa sjukdomar (Papulosquamous disorders) | L40-L45 | 696-697 696 Psoriasis och liknande sjukdomstillstånd 697 Lichen |
| Urtikaria och erytematösa tillstånd (Urticaria and erythema) | L50-L54 | 695A, 695B, 695C, 695W, 695X, 708 695A Toxiskt erytem 695B Erythema multiforme 695C Erythema nodosum 695W Andra erytematösa tillstånd 695X Erytematösa tillstånd, ospecificerade 708 Nässelfeber |
| Sjukdomar i hår, hårfolliklar, naglar, talgkörtlar och svettkörtlar (Disorders of skin appendages) | L60-L75 | 695D, 703-706 695D Rosacea 703 Sjukdomar i naglar 704 Sjukdomar i hår och hårfolliklar 705 Sjukdomar i svettkörtlarna 706 Sjukdomar i talgkörtlarna |
| Andra sjukdomar i hud och underhud (Other disorders of the skin and subcutaneous tissue) | L80-L99 | 695E, 700-702, 707, 709 695E Lupus erythematosus discoides 700 Liktornar och hudförhårdnader 701 Annan hypertrofisk och atrofisk hudsjukdom 702 Andra dermatoser 707 Kroniskt hudsår 709 Andra sjukdomar i hud och underhud |
| Symtom och sjukdomstecken från huden och underhuden (Symptoms and signs involving the skin and subcutaneous tissue) | R20-R23 | 782A, 782B, 782C, 782F, 782G, 782H, 782W, 782X 782A Störning av känsel i huden 782B Rodnad och andra icke specifika hudutslag 782C Lokaliserad ytlig svullnad eller knuta 782F Cyanos 782G Blekhet och rodnad 782H Spontana punktformiga blödningar 782W Förändringar i hudstrukturen 782X Andra symtom från huden och andra stödjevävnader |
|  |  |  |
| **13. Sjukdomar i muskuloskeletala systemet och bindväven (Diseases of the musculoskeletal system and connective tissue)** | | |
| Inflammatoriska polyartriter (Inflammatory polyarthropathies) | M05-M14 | 274, 712, 713, 714 274 Gikt 712 Kristallartropatier 713 Artropati förenad med andra sjukdomar som klassificeras annorstädes 714 Reumatoid artrit och liknande former |
| Artros (Arthrosis) | M15-M19 | 715 715 Artros och liknande sjukdomar |
| Andra ledsjukdomar (Other joint disorders) | M20-M25 | 716, 717, 719, 734-736 716 Andra och icke specificerade artropatier 717 Sjukdomar i meniskerna och i andra ledbrosk i knäleden 719 Andra och ospecificerade sjukdomar i lederna 734 Plattfot 735 Förvärvade deformiteter i tår 736 Andra förvärvade deformiteter av extremiteter |
| Inflammatoriska systemsjukdomar (Systemic connective tissue disorders) | M30-M36 | 446, 710, 711C, 725 446 Polyarteritis nodosa och därmed besläktade tillstånd 710 Systemiska bindvävssjukdomar 711C Artropati vid Behcets syndrom 725 Reumatisk polymyalgi |
| Ryggsjukdomar (Dorsopathies) | M40-M54 | 720-724, 737 Ryggåkommor (720-724) 737 Onormal ryggradskrökning |
| Sjukdomar i mjukvävnader (Soft tissue disorders) | M60-M79 | 726-729 726 Mjukdelsreumatism och liknande syndrom 727 Andra sjukdomar i ledhinnor, senor och slemsäckar 728 Sjukdomar i muskler, ligament 729 Andra sjukdomar i mjukvävnader |
| Sjukdomar i benvävnad och broskvävnad (Osteopathies and chondropathies) | M80-M94 | 730-733 730 Osteomyelit (benröta) och periostit 731 Deformerande beninflammation och bensjukdom förenad med T sjukdomar som klassificeras annorstädes 732 Samtidig ben- och brosksjukdom 733 Andra sjukdomar i ben och brosk |
| Andra sjukdomar i muskuloskeletala systemet och bindväven (Other disorders of the musculoskeletal system and connective tissue) | M95-M99 | 738 738 Annan förvärvad deformitet |
|  |  |  |
| **14. Sjukdomar i urin- och könsorganen (Diseases of the genitourinary system)** | |  |
| Glomerulussjukdomar (Glomerular diseases) | N00-N08 | 580-582, 583A, 583B, 583C 580 Akut glomerulonefrit 581 Nefrotiskt syndrom 582 Kronisk glomerulonefrit 583A Nefrit eller nefropati, ej specificerad som akut eller kronisk, med proliferativa glomerulusförändringar 583B Nefrit eller nefropati, med membranösa glomerulusförändringar 583C Nefrit eller nefropati, med både membranösa och proliferativa glomerulusförändringar |
| Tubulo-interstitiella njursjukdomar (Renal tubulo-interstitial diseases) | N10-N16 | 590-591, 593D, 593E, 593F, 593H, 599G 590 Njurinfektion 591 Hydronefros 593D Striktur av eller knickbildning på uretär 593E Annan uretärobstruktion 593F Hydrouretär 593H Vesikouretär reflux 599G Icke specificerat urinvägshinder |
| Njursvikt (Renal failure) | N17-N19 | 583G, 583H, 584-586 583G Nefrit eller nefropati, med skada av typen kortikal nekros 583H Nefrit eller nefropati,  med skada av typen medullär nekros 584 Akut njursvikt 585 Kronisk njursvikt 586 Icke specificerad njursvikt |
| Sten i urinvägarna (Urolithiasis) | N20-N23 | 788A, 592, 594 788A Njurkolik 592 Sten i njure och uretär 594 Sten i de nedre urinvägarna |
| Andra sjukdomar i njure och urinledare (Other disorders of kidney and ureter) | N25-N29 | 583E, 583W, 583X, 587-589, 593A, 593B, 593C, 593W, 593X 583E Nefrit eller nefropati, ej specificerad som akut eller kronisk, med snabbt progressivt förlopp 583W Nefrit eller nefropati, ej specificerad som akut eller kronisk, med annan specificerad patologisk-anatomisk njurförändring 583X Nefrit eller nefropati, ej specificerad som akut eller kronisk, med icke specificerad patologisk-anatomisk njurförändring 587 Icke specificerad njurskleros 588 Sjukdomar orsakade av nedsatt njurfunktion 589 Liten njure av okänd orsak 593A Nefroptos 593B Hypertrofi av njure 593C Njurcysta, förvärvad 593W Andra specificerade sjukdomar i njure och uretär 593X Andra sjukdomar i njure och uretär, ospecificerade 583X Nefrit eller nefropati, ej specificerad som akut eller kronisk, med icke specificerad patologisk-anatomisk njurförändring |
| Andra sjukdomar i urinorganen (Other diseases of urinary system) | N30-N39 | 593G, 595-597, 598A, 598B, 598W, 598X, 599A, 599B, 599C, 599D, 599E, 599F, 599H, 599W, 599X 593G Postural proteinuri 595 Cystit 596 Andra sjukdomar i urinblåsan 597 Icke venerisk uretrit 598A Uretrastriktur beroende på infektion 598B Traumatisk uretrastriktur 598W Annan specificerad uretrastriktur 598X Uretrastriktur, ospecificerad 599A Urinvägsinfektion med icke specificerad 599B Uretrafistel 599C Uretradivertikel 599D Uretrakarunkel 599E Falsk uretragång 599F Prolaps av uretraslemhinnan 599H Hematuri (blod i urinen) 599W Annan specificerad sjukdom i uretra och urinvägarna 599X Andra sjukdomar i uretra och urinvägarna, ospecificerade |
| Sjukdomar i de manliga könsorganen (Diseases of male genital organs) | N40-N51 | 600-608 Sjukdomar i de manliga könsorganen (600-608) |
| Sjukdomar i bröstkörtel (Disorders of breast) | N60-N64 | 610-611 Sjukdomar i bröstkörteln (610-611) |
| Inflammatoriska sjukdomar i de kvinnliga bäckenorganen (Inflammatory diseases of female pelvic organs) | N70-N77 | 614-616, 621F Inflammatoriska sjukdomar i de kvinnliga bäckenorganen (614-616) 621F Intrauterina synekier |
| Andra sjukliga tillstånd i urin- och könsorganen (Other disorders of the genitourinary system) | N99-N99 | 598C 598C Postoperativ uretrastriktur |
| Symtom och sjukdomstecken från urinorganen (Symptoms and signs involving the urinary system) | R30-R39 | 788B, 788C, 788D, 788E, 788F, 788G, 788H, 788W, 788X 788B Smärtor vid vattenkastning 788C Urinretention 788D Urininkontinens 788E Täta urintömningar och polyuri 788F Oliguri och anuri 788G Andra rubbningar i urineringen 788H Flytning från uretra 788W Urinutträngning i vävnaderna 788X Andra symtom från urinorganen |
| Onormala fynd vid urinundersökning utan diagnos (Abnormal findings on examination of urine, without diagnosis) | R80-R82 | 791 791 Ospecifika fynd vid urinundersökning |
|  |  |  |
|  |  |  |
| **18. Symtom, sjukdomstecken och onormala kliniska fynd och laboratoriefynd som ej klassificeras på annan plats (Symptoms, signs and abnormal clinical and laboratory findings, not elsewhere classified)** |  |  |
| Allmänna symtom och sjukdomstecken (General symptoms and signs) | R50-R69 | 780C, 780D, 780G, 780H, 780W, 780X, 781F, 782D, 783, 784A, 785F, 785G, 796E, 796X, 797, 799D, 799E, 799W, 799X 780C Svimning 780D Kramper 780G Feber av okänd orsak 780H Trötthet 780W Hyperhidros 780X Andra allmänna symtom, ospecificerade 781F Klubbfingrar 782D Ödem 783A Anorexi 783B Onormal viktökning 783C Onormal viktförlust 783D tproblem och vanskötsel 783E Utebliven förväntad normal fysiologisk  783F Polydipsi 783G Polyfagi 783X Andra symtom avseende nutrition, ämnesomsättning och utveckling 784A Huvudvärk 785F Chock utan uppgift om skada 785G Lymfkörtelförstoring 796E Andra onormala kliniska fynd 796X Andra ospecifika onormala fynd 797 Senilitet utan uppgift om psykos 799D Ospecificerad svaghet 799E Kakexi 799W Andra ofullständigt definierade tillstånd 799X Annan okänd och ospecificerad orsak |
| Onormala fynd vid undersökning av andra kroppsvätskor, substanser och vävnader, utan diagnos (Abnormal findings on examination of other body fluids, substances and tissues, without diagnosis) | R83-R89 | 792, 795A, 795B, 795C, 795D, 795E 792 Ospecifika onormala fynd i andra kroppssubstanser 795A Ospecifika onormala Papanicolaou-cervixutstryk 795B Ospecifika onormala Papanicolaou-utstryk av annat ursprung 795C Ospecifika onormala fynd vid kromosomanalys 795D Ospecifika positiva odlingsfynd 795E Andra ospecifika onormala histologiska fynd |
| Onormala fynd vid radiologisk diagnostik och vid funktionsundersökning utan diagnos (Abnormal findings on diagnostic imaging and in function studies, without diagnosis) | R90-R94 | 793-794 793 Ospecifika onormala fynd vid radiologisk och annan T undersökning av kroppsstrukturer 794 Ospecifika onormala resultat av funktionsundersökningar |
| Ofullständigt definierade och okända orsaker till död (Ill-defined and unknown causes of mortality) | R95-R99 | 798 798 Plötslig död utan känd orsak |
|  |  |  |
| **19. Skador, förgiftningar och vissa andra följder av yttre orsaker (Injury, poisoning and certain other consequences of external causes)** | | |
| Skador (Injury) | S00-T32 | 800-904, 910-957 800-829 Frakturer  830-839 Luxationer (urledvridningar) 840-848 Distorsioner (vrickningar och stukningar) och rupturer av muskler och senor 850-854 Skador på huvudet utan skallfraktur 860-869 Inre skador i bröstet, buken och bäckenet 870-897 Sårskador 900-904 Skador på blodkärl 910-919 Ytliga skador 920-924 Krosskador med intakt hud 925-929 Klämskador 930-939 Främmande kropp som trängt in genom naturlig öppning 940-949 Brännskador 950-957 Nerv- och ryggmärgsskador |
| Förgiftning (Poisoning) | T36-T65 | 960-989 960-979 Förgiftningar av droger, läkemedel och biologiska substanser 980-989 Toxisk effekt av substanser i huvudsakligen icke-medicinsk användning |
|  |  |  |
| **20. Yttre orsaker till sjukdom och död (External causes of morbidity and mortality)** | |  |
| Avsiktligt självdestruktiv handling (Intentional self-harm) | X60-X84 | E950-E959 E950-E959 Självmord, självmordsförsök och annan självtillfogad skada |
| Komplikationer vid medicinsk vård och kirurgiska åtgärder (Complications of medical and surgical care) | Y40-Y84 | E87, E947 E870-E879 Missöden och komplikationer vid medicinsk åtgärd E947 Ogynnsam effekt av droger, läkemedel och biologiska substanser i terapeutiskt bruk |
|  |  |  |
| **Medfödda tillstånd (Congenital conditions)** | F71.1 Medelsvår psykisk utvecklingsstörning - betydande beteendestörning som kräver vård eller behandling (Moderate mental retardation, significant impairment of behaviour requiring attention or treatment) F72 Svår psykisk utvecklingsstörning (Severe mental retardation) F73 Grav psykisk utvecklingsstörning (Profound mental retardation) F78.2,1 Annan psykisk utvecklingsstörning (Other mental retardation) F79.1 Ospecificerad psykisk utvecklingsstörning - betydande beteendestörning som kräver vård eller behandling (Unspecified mental retardation : significant impairment of behaviour requiring attention or treatment) F83 Blandade specifika utvecklingsstörningar (Mixed specific developmental disorders) F84 Genomgripande utvecklingsstörningar (Pervasive developmental disorders) G80 Cerebral pares (Cerebral palsy) Q01-03 Anencefali, encefalocele och microcefali (Anencephaly and similar malformations, encephalocele and microcephaly) Q05 Spina bifida (Spina bifida) Q20-26 Medfödda missbildningar av hjärtat och stora kärl (Congenital malformations of heart and great blood vessels) Q90 Downs syndrom (Down syndrome) | 299, 315W, 318A, 318B, 318C, 343, 741, 742A, 742B, 742D, 745, 746, 747A, 747B, 747C, 747D, 747E 318A Måttlig psykisk utvecklingsstörning 318B Svår psykisk utvecklingsstörning 318C Djup psykisk utvecklingsstörning 315W Andra specifika störningar i utvecklingen 299A Infantil autism 299B Desintegrativ psykos 299W Annan specificerad barndomspsykos 299X Barndomspsykos, ospecificerad 343 Cerebral pares 742A Encefalocele 742B Mikrocefali 742D Medfödd hydrocefalus 741 Spina  745 Missbildningar vid hjärtats anläggning och hjärtskiljeväggens slutning 746 Andra medfödda hjärtmissbildningar 747A Bestående ductus arteriosus 747B Coartatio aortae 747C Andra missbildningar av aorta 747D Missbildningar av lungartären 747E Missbildningar av de stora venerna |

## eTable 3, Educational Status in Sepsis Cohort at 20, 25 and 30 years of age, Stepwise Logistic Regression Analysis

|  |  |  | **Crude** | | | **Adjusted^a^** | | |
| --- | --- | --- | --- | --- | --- | --- | --- | --- |
| **Group** | **Split** | **Variable** | **N** | **OR (95% CI)** | **P-value** | **N** | **OR (95% CI)** | **P-value** |
| **20 years of age** | Compulsory school education 9 years or less versus at least upper secondary education 2 years | Sepsis Cohort | 53 437 | 1.07 (1.03 - 1.13) | 0.002 | 53 362 | 1.13 (1.07 - 1.18) | <0.001 |
|  | Upper secondary education 2 years or less, versus at least upper secondary education 3 years | Sepsis Cohort | 53 437 | 1.07 (1.03 - 1.12) | 0.001 | 53 362 | 1.13 (1.08 - 1.18) | <0.001 |
|  | Upper secondary education 3 years or less, versus at least post-secondary education shorter than 3 years | Sepsis Cohort | 53 437 | 1.08 (1.02 - 1.14) | 0.012 | 53 362 | 1.11 (1.04 - 1.18) | <0.001 |
| **25 years of age** | Compulsory school education 9 years or less, versus at least upper secondary education 2 years | Sepsis Cohort | 38 263 | 1.08 (1.02 - 1.15) | 0.013 | 38 203 | 1.10 (1.03 - 1.18) | 0.003 |
|  | Upper secondary education 2 years or less, versus at least upper secondary education 3 years | Sepsis Cohort | 38 263 | 1.10 (1.05 - 1.16) | <0.001 | 38 203 | 1.13 (1.07 - 1.20) | <0.001 |
|  | Upper secondary education 3 years or less, versus at least post-secondary education shorter than 3 years | Sepsis Cohort | 38 263 | 1.09 (1.04 - 1.13) | <0.001 | 38 203 | 1.10 (1.05 - 1.15) | <0.001 |
|  | Post-secondary education shorter than 3 years, versus post-secondary education 3 years or more | Sepsis Cohort | 38 263 | 1.05 (1.00 - 1.11) | 0.056 | 38 203 | 1.07 (1.01 - 1.12) | 0.021 |
| **30 years of age** | Compulsory school education 9 years or less, versus at least upper secondary education 2 years | Sepsis Cohort | 22 642 | 1.20 (1.10 - 1.31) | <0.001 | 22 607 | 1.23 (1.13 - 1.34) | <0.001 |
|  | Upper secondary education 2 years or less, versus at least upper secondary education 3 years | Sepsis Cohort | 22 642 | 1.16 (1.08 - 1.24) | <0.001 | 22 607 | 1.19 (1.11 - 1.28) | <0.001 |
|  | Upper secondary education 3 years or less, versus at least post-secondary education shorter than 3 years | Sepsis Cohort | 22 642 | 1.09 (1.03 - 1.15) | 0.002 | 22 607 | 1.11 (1.05 - 1.17) | <0.001 |
|  | Post-secondary education shorter than 3 years, versus post-secondary education 3 years or more | Sepsis Cohort | 22 642 | 1.09 (1.03 - 1.16) | 0.003 | 22 607 | 1.11 (1.04 - 1.18) | <0.001 |
| ^a^Adjusted for age, sex and comorbidities | | | | | | | | |

## eTable 4, Charlson Comorbidity Index, Swedish Adaption

| **Charlson Comorbidity Index** | **ICD-10-SE** | **ICD 9** |
| --- | --- | --- |
| **Myocardial infarction** | I21 (Akut hjärtinfarkt, acute myocardial infarction), I22 (Reinfarkt, re-infarction), I25.2 (Gammal hjärtinfarkt, old myocardial infarction) | 410 (Akut hjärtinfarkt, acute myocardial infarction), 412 (Gammal hjärtinfarkt, old myocardial infarction) |
| **Congestive heart failure** | I11.0 (Hypertoni med hjärtsjukdom, Hypertensive heart disease) I13.0 (Hypertoni med hjärt- och njursjukdom, Hypertensive heart and kidney disease) I13.2 (Hypertensiv hjärt- och njursjukdom med både hjärtsvikt och njursvikt, Hypertensive heart and kidney disease with both (congestive) heart failure and kidney failure) I25.5 (Ischemisk kardiomyopati, Ischaemic cardiomyopathy) I42.0 (Dilaterad kardiomyopati, Dilated cardiomyopathy) I42.6 (Alkoholkardiomyopati, Alcoholic cardiomyopathy) I42.7 (Kardiomyopati orsakad av läkemedel och annan yttre påverkan, Cardiomyopathy due to drugs and other external agents) I42.8 (Andra specificerade kardiomyopatier, Other cardiomyopathies) I42.9 (Kardiomyopati, ospecificerad, Cardiomyopathy, unspecified) I43 (Hjärtmuskelsjukdom vid sjukdomar som klassificeras annorstädes, Cardiomyopathy in infectious and parasitic diseases classified elsewhere) I50 (Kronisk hjärtinsufficiens, Congestive heart failure) | 402A (Malign hypertoni med hjärtsjukdom, malignant hypertension with heart disease) 402B (Benign hypertoni med hjärtsjukdom, benign hypertensive heart disease) 402X (Hypertoni, ospecificerad, med hjärtsjukdom, unspecfied hypertensive heart disease) 404A (Malign hypertoni med hjärt- och njursjukdom, malignant hypertensive heart and kidney disease) 404B (Benign hypertoni med hjärt- och njursjukdom med hjärtsvikt och njursvikt, benign hypertensive heart and kidney disease with heart failure and kidney failure) 404X (Hypertoni, ospecificerad, med hjärt- och njursjukdom, unspecified Hypertensive heart and kidney disease) 425E (Annan primär kardiomyopati, Other primary cardiomyopathies) 425F (Alkoholkardiomyopati, Alcoholic cardiomyopathy) 425H (Kardiomyopati vid nutritions- och ämnesomsättningsrubbning, Nutritional and metabolic cardiomyopathies) 425W (Kardiomyopati vid andra sjukdomar, Cardiomyopathy in other diseases classified elsewhere) 425X (Sekundär kardiomyopati, icke specificerad, Secondary cardiomyopathy, unspecified) 428 (Hjärtsvikt, heart failure) |
| **Peripheral vascular disease** | I70 (Ateroskleros, Atherosclerosis) I71 (Aortaaneurysm, Aortic aneurysm and dissection) I73.1 (Thrombangiitis obliterans, Thromboangiitis obliterans) I73.8 (Andra specificerade sjukdomar i perifera kärl, Other specified peripheral vascular diseases) I73.9 (Perifer vaskulär sjukdom, ospecificerad, Peripheral vascular disease, unspecified) I77.1 (Artärstriktur, Stricture of artery) I79.0 (Aneurysm i aorta vid sjukdomar som klassificeras annorstädes, Aneurysm of aorta in diseases classified elsewhere) I79.2 (Perifer angiopati vid sjukdomar som klassificeras annorstädes, Peripheral angiopathy in diseases classified elsewhere) K55 (kärlsjukdomar i tarmen, vascular disorders of the intestine) | 440 (Ateroskleros, Atherosclerosis) 441 (Aortaaneurysm, aortic aneurysm) 443B (Thrombangiitis obliterans, Thromboangiitis obliterans) 443X (annan sjukdom i perifera kärl, other peripheral vascular disease) 447B (artärstenos, stricture of artery) 557 (kärlsjukdomar i tarmen, vascular disorders of the intestine) |
| **Cerebrovascular disease** | G45 (Övergående cerebral ischemi (Otillräcklig blodtillförsel till hjärnan) och besläktade syndrom, Transient cerebral ischaemic attack and related syndromes) I60 (Subaraknoidalblödning, Subarachnoid hemorrhage) I61 (Hjärnblödning, Intracerebral hemorrhage) I62 (Annan icke traumatisk intrakraniell blödning, Other nontraumatic intracranial hemorrhage) I63 (Cerebral infarkt, Cerebral infarction) I64 (Akut cerebrovaskulär sjukdom ej specificerad som blödning eller infarkt, Stroke, not specified as haemorrhage or infarction) I67 (Andra cerebrovaskulära sjukdomar, Other cerebrovascular diseases) I69 (Sena effekter av cerebrovaskulär sjukdom, late complications from cerebrovascular disease) | 430 (Subaraknoidalblödning, subarachnoid hemorrhage) 431 (Hjärnblödning, cerebral hemorrhage) 432 (Annan och ospecificerad intrakraniell blödning, other and unspecified intracranial hemorrhage) 433 (Ocklusion och stenos av precerebrala artärer, occlusion and stenosis of the precerebral arteries) 434 (Ocklusion av cerebrala artärer, occlusion of the cerebral arteries) 435 (Spasm i cerebralartär, spasm of cerebral artery) 436 (Akut ofullständigt preciserad cerebrovaskulär sjukdom, Acute incomplete cerebrovascular disease) 437 (Annan och ofullständigt preciserad cerebrovaskulär sjukdom, Other and incompletely specified cerebrovascular disease) 438 (Sena effekter av cerebrovaskulär sjukdom, Late effects of cerebrovascular disease) |
| **Chronic obstructive pulmonary disease (COPD)** | J43 (Lungemfysem, Emphysema) J44 (Kroniskt obstruktiv lungsjukdom, Other chronic obstructive pulmonary disease) | 491 (Kronisk luftrörskatarr, chronic bronchitis) 492 (Lungemfysem, emphysema) 496 (Kronisk obstruktiv lungsjukdom som ej klassificeras annorstädes, Chronic obstructive pulmonary disease that is not classified elsewhere) |
| **Other chronic pulmonary diseas** | J41 (Kronisk bronkit utan luftvägsobstruktion, Chronic bronchitis without airway obstruction) J42 (Icke-specificerad kronisk bronkit, unspecified chronic bronchitis) J45 (Astma, asthma) J46 (Akut svår astma, Acute severe asthma) J47 (Bronkiektasier, Bronchiectasis) J60 (Pneumokonios (Dammlunga) orsakad av stenkolsdamm, Coalworker’s pneumoconiosis) J61 (Pneumokonios (Dammlunga) orsakad av asbest och andra mineralfibrer, Pneumoconiosis due to asbestos and other mineral fibres) J62 (Pneumokonios (Dammlunga) orsakad av damm innehållande kisel, Pneumoconiosis due to dust containing silica) J63 (Pneumokonios (Dammlunga) orsakad av annat oorganiskt damm, Pneumoconiosis due to other inorganic dusts) J64 (Icke specificerad pneumokonios (Dammlunga), Unspecified pneumoconiosis) J65 (Pneumokonios (Dammlunga) förenad med tuberkulos, Pneumoconiosis associated with tuberculosis) J66 (Luftvägssjukdom orsakad av specificerat organiskt damm, Airway disease due to specific organic dust) J67 (Hypersensitivitetspneumonit (spridda, icke infektionsbetingade inflammatoriska förändringar i lungorna) orsakad av organiskt damm, Hypersensitivity pneumonitis due to organic dust) J68 (Sjukliga tillstånd i lungorna orsakade av kemikalier, gaser, rök och ånga, Respiratory conditions due to chemicals, gases, fumes and vapours) J69 (Pneumonit orsakad av fast eller flytade ämne, pneumonitis due to solid or fluid substance) J70 (Sjukliga tillstånd i lungorna av andra yttre orsaker, Respiratory conditions due to other external agents) | 490 (Bronkit – akut eller kronisk, Bronchitis, not specified as acute or chronic) 493 (Astma, asthma) 494 (Bronkiektasier, Bronchiectasis) 495 (Extrinsic allergic alveolitis, Hypersensitivitetspneumonit) 500 (Pneumokonios, pneumoconiosis) 501 (Asbestos, Asbestosis) 502 (Pneumokonios (Dammlunga) orsakad av kisel och andra kiselföreningar än asbest, Pneumoconiosis due to other silica or silicates) 503 (Pneumokonios (Dammlunga) orsakad av annat oorganiskt damm, Pneumoconiosis due to other inorganic dust) 504 (Lungsjukdom orsakad av inandning av annat damm, Pneumopathy due to inhalation of other dust) 505 (Icke specificerad pneumokonios (Dammlunga), Pneumoconiosis, unspecified) 506 (Sjukliga tillstånd i luftvägarna orsakade av rök eller ånga, Respiratory conditions due to fumes and vapours) 507 (Pneumonit orsakad av föda eller uppkräkt maginehåll, pneumonitis due to food or vomits) 508 (Lungmanifestationer av yttre orsaker, Pulmonary conditions due to external agents) 516 (Postinflammatorisk lungfibros; post- inflammatory lung fibrosis) 517 (Lungpåverkan vid annan sjukdom t.ex. reumatisk sjukdom och sarcoidos; pulmonary disease due to other disease including rheumatic conditions and sarcoidosis) |
| **Rheumatic disease** | M05; M06, M12.3 (Reumatoid artrit, rheumatoid arthritis) M07.0–3 (Psoriasis-artrit, psoriatic arthritis) M08 (Juvenil idiopatisk artrit, juvenile idiopathic arthritis) M13 (Polyartrit, poly arthritis) M30 (Polyarteritis nodosa) M31.3-M31.6 (Andra nekrotiserande vaskulopatier, Necrotizing vasculitides including giant cell arteritis) M32 (SLE) M33 (Dermatopolymyosit, Dermato- and polymyositis) M34 (Systemisk skleros, Systemic sclerosis) M35.0 (Sjögrens syndrome, Sjögren syndrome) M35.1 (Andra overlap-syndrom, Other overlap syndromes) M35.3 (Polymyalgia rheumatica, Polymyalgia rheumatica) M45-46 (Ankyloserande spondylit, ankylosing spondylitis) | 446 (Polyarteritis nodosa) 696A (psoriasis-artrit, psoriatic arthritis) 710A-E (SLE, sklerodermi, Sjögrens syndrom, dermatomyosit, polymyosit; SLE, scleroderma, sicca syndrome, dermatomyositis and polymyositis) 714 (Reumatoid artrit, rheumatoid arthritis including juvenile idiopathic arthritis) 719D (Palindrom rheumatoid artrit; palindromic rheumatoid arthritis) 720 (Ankyloserande spondylit, ankylosing spondylitis) 725 (Polymyalgia rheumatica, temporal arteritis) |
| **Dementia** | F00 (Demens vid Alzheimers sjukdom, Dementia in Alzheimer’s disease) F01 (Vaskulär demens, Vascular dementia) F02 (Demens vid andra sjukdomar som klassificeras annorstädes, Dementia in other diseases classified elsewhere) F03 (Ospecificerad demens, Unspecified dementia) F05.1 (Delirium med underliggande demenssjukdom, Delirium superimposed on dementia) G30 (Alzheimers sjukdom, Alzheimer’s disease) G31.1 (Senil degeneration av hjärnan som ej klassificeras annorstädes, Senile degeneration of brain, not elsewhere classified) G31.9 (Degenerativ sjukdom i nervsystemet, degenerative disease of the nervous system) | 290 (Olika typer av demens inklusive alzheimers, various forms of dementia including alzheimer’s disease) 294B (Demens på grund av somatisk sjukdom; dementia due to somatic disease) 331A-C, 331X (Presenil och senil degeneration inclusive Pick’s sjukdom; presenile/senile dementia including Pick’s disease) |
| **Hemiplegia, tetraplegia** | G11.4 (Hereditär spasisk paraplegi, hereditary spastic paraplegia) G80 (Cerebral pares, cerebral palsy) G81 (Ensidig förlamning, hemiplegia) G82 (parapares/tetrapares, paraplegia and tetraplegia) G83.0-G83.3 (Andra förlamningssyndrom, Monoplegia to tetraplegia) G83.8 (Andra specificerade förlamningssyndrom, tetraplegia) | 342 (Ensidig förlamning Hemiplegia) 343 (Cerebral pares, cerebral palsy) 344A-F (Paralysis cerebralis alia, Andra förlamningssyndrom från monoplegi till tetraplegi, Monoplegia ranging to tetraplegia) |
| **Diabetes** | E10.0 (Insulinberoende diabetes med koma, Diabetes mellitus with coma) E10.1 (Insulinberoende diabetes med ketoacidos, Diabetes mellitus with ketoacidosis) PLUS other forms of diabetes without End-stage organ damage E11.0-E11.1 E12.0-E12.1 E13.0-E13.1 E14.0-E14.1 | 250A (Diabetes, diabetes mellitus) 250B (Diabetes med ketoacidos, diabetes with ketoacidosis) 250C (Diabetes med koma, diabetes with coma) |
| **Diabetes with end organ damage** | E10.2 (Insulinberoende diabetes med njurkomplikationer, Diabetes mellitus renal complications) E10.3 (Insulinberoende diabetes med ögonkomplikationer, Diabetes mellitus with eye complications) E10.4 (Insulinberoende diabetes med neurologiska komplikationer, Diabetes mellitus with neurological complications) E10.5 (Insulinberoende diabetes med perifera cirkulationsrubbningar, Diabetes mellitus with peripheral vascular complications) E10.7 (Insulinberoende diabetes med multipla komplikationer, Diabetes mellitus with multiple complications) (AND (other forms of diabetes with same End- stage organ damage)) E11.2-E11.7 E12.2-E12.7 E13.2-E13.7 E14.2-E14.7 | N03.2-N03.7 (Kronisk glomerulonefrit – ej minimal change eller fokal, Chronic glomerulonephritis – not minimal change or focal) N05.2-N05.7 (Icke-specificerad glomerulonefrit – ej minimal change eller fokal, Unspecified glomerulonephritis – not minimal change or focal) N11 (Kronisk pyelonefrit, chronic pyelonephritis) N18 (Kronisk njursvikt, Chronic kidney failure) N19 (Njursvikt, icke specificerad som akut eller kronisk, Unspecified kidney failure) N25.0 (Renal osteodystrofi orsakade av nedsatt funktion i njurtubuli, Renal osteodystrophy resulting from impaired renal tubular function) I12.0 (Hypertensiv njursjukdom med njursvikt, Hypertensive kidney disease with kidney failure) I13.1 (Hypertensiv hjärt- och njursjukdom med njursvikt, Hypertensive heart and kidney disease with kidney failure) Q61.1-Q61.4 (Autosomal polycystisk njursjukdom, autosomal polycystic kidney disease) Z49 (Förberedande vård för dialys/ Extrakorporeal D./Annan D, Preparatory care for dialysis, Extracorporeal/Other dialysis) Z94.0 (Njurtransplanterad, kidney transplant) Z99.2 (Beroende av njurdialys, need for kidney dialysis) |
| **Moderate or severe kidney disease** | N03.2-N03.7 (Kronisk glomerulonefrit – ej minimal change eller fokal, Chronic glomerulonephritis – not minimal change or focal) N05.2-N05.7 (Icke-specificerad glomerulonefrit – ej minimal change eller fokal, Unspecified glomerulonephritis – not minimal change or focal) N11 (Kronisk pyelonefrit, chronic pyelonephritis) N18 (Kronisk njursvikt, Chronic kidney failure) N19 (Njursvikt, icke specificerad som akut eller kronisk, Unspecified kidney failure) N25.0 (Renal osteodystrofi orsakade av nedsatt funktion i njurtubuli, Renal osteodystrophy resulting from impaired renal tubular function) I12.0 (Hypertensiv njursjukdom med njursvikt, Hypertensive kidney disease with kidney failure) I13.1 (Hypertensiv hjärt- och njursjukdom med njursvikt, Hypertensive heart and kidney disease with kidney failure) Q61.1-Q61.4 (Autosomal polycystisk njursjukdom, autosomal polycystic kidney disease) Z49 (Förberedande vård för dialys/ Extrakorporeal D./Annan D, Preparatory care for dialysis, Extracorporeal/Other dialysis) Z94.0 (Njurtransplanterad, kidney transplant) Z99.2 (Beroende av njurdialys, need for kidney dialysis) | 403A (Malign hypertoni med njursjukdom, malignant hypertensive kidney disease) 403B (Benign hypertoni med njursjukdom, Hypertensive kidney disease) 403X (Hypertoni, ospecificerad, med njursjukdom, Hypertensive kidney disease) 582 (Kronisk glomerulonefrit, chronic glomerulonephritis) 583 (Nefrit eller nefropati med påvisade anatomisk/patologiska förändrignar, nephritis or nephropathy with confirmed anatomical or histopathological changes) 585 (Kronisk njursvikt, chronic kidney failure) 586 (Icke specificerad njursvikt, unspecified kidney failure) 588A (Renal osteodystrofi, Renal osteodystrophy) V42A (Njurtransplantation, kidney transplantation) V45B (Behov av återkommande njurdialys, Kidney dialysis) V56 (Eftervård med återkommande dialys, Aftercare involving intermittent dialysis) |
| **Mild liver disease** | B15-B19 (Virushepatit, viral hepatitis) K70.3 (Leversjukdom orsakad av alkohol, ospecificerad,Alcoholic liver disease, unspecified) K73 (Kronisk hepatit som ej klassificeras annorstädes, Chronic hepatitis, not elsewhere classified) K74.6 (Leverfibros och levercirros, Fibrosis and liver cirrhosis) K70.3 (Levercirros orsakad av alkohol, Alcoholic liver cirrhosis) K75.4 (Autoimmune hepatit, autoimmune hepatitis) | 070 (Virushepatit, viral hepatitis) 571C (Levercirros orsakad av alkohol, Alcoholic liver cirrhosis) 571E (Kronisk hepatit, Chronic hepatitis) 571F (Levercirros utan uppgift om alkohol som orsak, Liver cirrhosis without mention of alcohol) 573 (Hepatit UNS, unspecific hepatitis) |
| **Moderate or severe liver disease** | R18 (+Any code for mild liver disease in combination with ascites) I85.0 (Esofagusvaricer med blödning, Oesophageal varices with bleeding) I85.9 (Esofagusvaricer utan blödning, Oesophageal varices without bleeding) I98.2 (Esofagusvaricer utan blöding vid sjukdomar som klassificeras annorstädes, Oesophageal varices without bleeding, in diseases classified elsewhere) I98.3 (Esofagusvaricer med blödning vid sjukdomar som klassificeras annorstädes, Oesophageal varices with bleeding, diseases classified elsewhere) | 789F (+Any code for mild liver disease in combination with ascites) 456A (Esofagusvaricer med blödning, Oesophageal varices with bleeding) 456B (Esofagusvaricer utan uppgift om blödning, Oesophageal varices without mention of bleeding) 456C (Esofagusvaricer vid levercirrhos, Oesophageal varices and liver cirrhosis) 572D (Portahypertension, Portal hypertension) 572E (Hepatorenalt syndrom, Hepatorenal syndrome) 572C (Leverencefalopati, Hepatic Encephalopathy |
| **(Peptic) Ulcer disease** | K25 (Sår i magsäcken, Gastric ulcer) K26 (Sår i tolvfingertarmen, Duodenal ulcer) K27 (Sår i magsäcken eller tolvfingertarmen utan angiven lokalisation, Peptic ulcer, site unspecified) K28 (Recidivsår efter gastroenterostomi (operation av magsäcken), Gastrojejunal ulcer) | 531 (Sår i magsäcken, Gastric ulcer) 532 (Sår i tolvfingertarmen, Duodenal ulcer) 533 (Sår på magsäck eller tolvfingertarm utan angiven lokalisation, Peptic ulcer - site unspecified) 534 (Recidivsår efter magsäcks-operation, Recurrent ulcer after gastric surgery) |
| **Any malignancy, including leukemia and lymphoma** | C00-C97, ej C87 | 140-208 |
| **Metastatic cancer** | C77 (Sekundär malign tumör (metastas) och icke specificerad malign tumör i Lymfkörtlar, Secondary and unspecified malignant neoplasm of the lymph nodes) C78 (Sekundär malign tumör (metastas) i andningsorganen och matsmältningsorganen, Secondary malignant neoplasm of the respiratory and digestive organs) C79 (Sekundär malign tumör (metastas) med andra lokalisationer, Secondary malignant neoplasm of other sites) C80 (Malign tumör utan specificerad lokalisation, Malignant neoplasm without specification of site) | 196 (Sekundär och icke specificerad malign tumör i lymfkörtlarna, Secondary and unspecified malignant neoplasm of the lymph nodes) 197 (Sekundär malign tumör i andningsorganen och matsmältningsorganen, Secondary malignant neoplasm of the respiratory and digestive organs) 198 (Sekundär malign tumör med andra specificerade lokalisationer, Secondary malignant neoplasm of other specified sites) 199A (Malign tumör utan specificerad lokalisation, Malignant neoplasm without specification of site) 199B (Annan lokalisation, other localisation) |
| **HIV/AIDS** | B20 (Sjukdom orsakad av humant immunbristvirus (HIV) tillsammans med infektions- och parasitsjukdom, Human immunodeficiency virus (HIV) disease resulting in infectious and parasitic disease) B21 (Sjukdom orsakad av humant immunbristvirus (HIV) tillsammans med maligna tumörer, Human immunodeficiency virus (HIV) disease resulting in malignant neoplasms) B22 (Sjukdom orsakad av humant immunbristvirus (HIV) tillsammans med andra specificerade sjukdomar, Human immunodeficiency virus (HIV) disease resulting in other specified diseases) B23 (Sjukdom orsakad av humant immunbristvirus (HIV) tillsammans med andra tillstånd, Human immunodeficiency virus (HIV) disease resulting in other conditions) B24 (Icke specificerad sjukdom orsakad av humant immunbristvirus (HIV), Unspecified human immunodeficiency virus (HIV) disease) F02.4 (Demens vid sjukdom orsakad av humant immunbristvirus (HIV), HIV dementia) O98.7 (HIV som komplicerande faktor hos gravid kvinna, HIV as complicating factor in pregnant woman) R75 (Positivt HIV-test, positive HIV test) Z11.4 (Riktad hälsokontroll avseende humant immunbristvirus (HIV), Health check-up for HIV) Z21.9 (Asymtomatisk infektion med humant immunbristvirus (HIV), asymptomatic HIV infection) Z71.1 (Rådgivning vid HIV, Human immunodeficiency virus (HIV) counseling) | 079J (Sjukdom orsakat av HTLV-III virus, disease caused by the HTLV-III virus) 279K (Humant immunbristvirus, Human immunodeficiency virus (HIV) disease) |

## eTable 5, Competing Risk Analysis of Mordbidity in Sepsis Cohort with Fine-Gray Subdistribution Hazard Model, with Death as Competing Risk

|  | **Crude** | | **Adjusted^a^** | |
| --- | --- | --- | --- | --- |
| **Variable** | **HR (95% CI)** | **P-value** | **HR (95% CI)** | **P-value** |
| **New diagnosis within 3 months of discharge after index hospitalization** |  | | | |
| Sepsis, n (%) | 6.72 (6.22 - 7.25) | <0.001 | 4.25 (3.92 - 4.61) | <0.001 |
| Infectious diseases, n (%) | 1.31 (1.26 - 1.35) | <0.001 | 1.18 (1.14 - 1.22) | <0.001 |
| Neoplasms, n (%) | 1.40 (1.23 - 1.60) | <0.001 | 0.93 (0.80 - 1.07) | 0.31 |
| Mental and behavioural disorders, n (%) | 0.80 (0.69 - 0.91) | 0.001 | 0.69 (0.59 - 0.79) | <0.001 |
| Diseases of the circulatory system, n (%) | 0.87 (0.82 - 0.93) | <0.001 | 0.83 (0.78 - 0.89) | <0.001 |
| Diseases of the respiratory system, n (%) | 1.20 (1.11 - 1.30) | <0.001 | 1.11 (1.02 - 1.21) | 0.016 |
| **New diagnosis 3-12 months after discharge after index hospitalization** |  | | | |
| Sepsis, n (%) | 2.55 (2.33 - 2.80) | <0.001 | 1.67 (1.51 - 1.84) | <0.001 |
| Infectious diseases, n (%) | 1.09 (1.06 - 1.12) | <0.001 | 1.07 (1.04 - 1.10) | <0.001 |
| Neoplasms, n (%) | 1.37 (1.22 - 1.53) | <0.001 | 0.90 (0.80 - 1.02) | 0.1 |
| Mental and behavioural disorders, n (%) | 0.92 (0.84 - 1.00) | 0.055 | 0.82 (0.75 - 0.90) | <0.001 |
| Diseases of the circulatory system, n (%) | 0.95 (0.89 - 1.00) | 0.07 | 0.90 (0.84 - 0.95) | <0.001 |
| Diseases of the respiratory system, n (%) | 1.20 (1.11 - 1.30) | <0.001 | 1.11 (1.02 - 1.21) | 0.016 |
| **New diagnosis 1-5 years after discharge after index hospitalization** |  | | | |
| Sepsis, n (%) | 1.67 (1.52 - 1.84) | <0.001 | 1.38 (1.26 - 1.52) | <0.001 |
| Infectious diseases, n (%) | 0.98 (0.96 - 1.01) | 0.17 | 0.98 (0.96 - 1.01) | 0.198 |
| Neoplasms, n (%) | 1.64 (1.50 - 1.79) | <0.001 | 1.07 (0.97 - 1.18) | 0.161 |
| Mental and behavioural disorders, n (%) | 1.03 (0.98 - 1.08) | 0.22 | 0.94 (0.90 - 0.99) | 0.014 |
| Diseases of the circulatory system, n (%) | 1.03 (0.98, 1.07) | 0.24 | 0.97 (0.93 - 1.02) | 0.23 |
| Diseases of the respiratory system, n (%) | 1.00 (0.97 - 1.03) | 0.77 | 1.05 (1.02 - 1.08) | 0.001 |
| ^a^Adjusted for age and comorbidities. | | | | |

## eTable 6, Age Stratification of Overall Survival, Cox Regression Analysis

|  | | **Crude** | | **Adjusted^a^** | |
| --- | --- | --- | --- | --- | --- |
| **Group** | **Cohort** | **Hazard Ratio (95% CI)** | **P-value** | **Hazard Ratio (95% CI)** | **P-value** |
| **Age at index hospitalization: 0-<1 years** | Sepsis Cohort | 1.23 (1.17-1.30) | <0.001 | 1.20 (1.13-1.26) | <0.001 |
| **Age at index hospitalization: 1-<7 years** | Sepsis Cohort | 1.76 (1.56-1.98) | <0.001 | 1.29 (1.14-1.47) | <0.001 |
| **Age at index hospitalization: 7-<13 years** | Sepsis Cohort | 1.73 (1.48-2.03) | <0.001 | 1.19 (1.00-1.41) | 0.050 |
| **Age at index hospitalization: 13-<18 years** | Sepsis Cohort | 1.34 (1.18-1.53) | <0.001 | 0.82 (0.71-0.95) | 0.006 |
| **Age at index hospitalization: 1-<18 years** | Sepsis Cohort | 1.57(1.46 - 1.70) | <0.001 | 1.08 (1.00 - 1.18) | 0.056 |
| **All hospitalizations** | Sepsis Cohort | 1.46 (1.40-1.53) | <0.001 | 1.17 (1.11-1.22) | <0.001 |
| a: Adjusted for comorbidities, and in age group 1-<18 adjusted for age | | | | | |

## eTable 7, Sensitivity Analyses of Overall Survival, Cox Regression Analysis

|  | | **Crude** | | **Adjusted^a^** | |
| --- | --- | --- | --- | --- | --- |
| **Group** | **Cohort** | **Hazard Ratio (95% CI)** | **P-value** | **Hazard Ratio (95% CI)** | **P-value** |
| **Index hospitalization in ICD-10-SE (from year 1997)** | Sepsis Cohort | 1.75 (1.66-1.85) | <0.001 | 1.36 (1.28-1.44) | <0.001 |
| **Index hospitalization in ICD-9 (until year 1996)** | Sepsis Cohort | 1.05 (0.97-1.13) | 0.208 | 0.88 (0.81-0.95) | 0.001 |
| **Excluding patients with cancer diagnosis** | Sepsis Cohort | 1.23 (1.17-1.29) | <0.001 | 1.18 (1.13-1.24) | <0.001 |
| **Index hospitalization from year 2001, with specialized outpatient care data available** | Sepsis Cohort | 1.76 (1.65-1.86) | <0.001 | 1.41 (1.32-1.50) | <0.001 |
| **All hospitalizations** | Sepsis Cohort | 1.46 (1.40-1.53) | <0.001 | 1.17 (1.11-1.22) | <0.001 |
| a: Adjusted for age and comorbidities | | | | | |

| eTable 8, Sensitivity Analysis of Morbidity Outcome, 0-<1 Years and 1-<18 Years, Logistic Regression, 0-5 Years After Discharge | | | | | | | |  |
| --- | --- | --- | --- | --- | --- | --- | --- | --- |
|  |  | | **Crude** | | | **Adjusted^a^** | | |
| **Variable** | **Control Cohort** | **Sepsis Cohort** | **OR (95% CI)** | | **P-value** | **OR (95% CI)** | | **P-value** |
| **New diagnosis within 3 months of discharge after index hospitalization** | | | | | | | | |
| **0-<1 years at inclusion** |  | | | | | | | |
| Sepsis, n (%) | 505 (0.7%) | 917 (2.3%) | 3.51 (3.15 - 3.92) | | <0.001 | 3.26 (2.92 - 3.65) | | <0.001 |
| Infectious diseases, n (%) | 7 622 (9.9%) | 4 405 (11%) | 1.11 (1.07 - 1.16) | | <0.001 | 1.09 (1.05 - 1.14) | | <0.001 |
| Neoplasms, n (%) | 381 (0.5%) | 182 (0.4%) | 0.91 (0.76 - 1.08) | | 0.285 | 0.86 (0.72 - 1.03) | | 0.111 |
| Mental and behavioural disorders, n (%) | 277 (0.4%) | 147 (0.4%) | 1.01 (0.82 - 1.23) | | 0.929 | 1.00 (0.82 - 1.22) | | 0.977 |
| Diseases of the circulatory system, n (%) | 2 231 (2.9%) | 1 033 (2.6%) | 0.88 (0.81 - 0.95) | | <0.001 | 0.87 (0.80 - 0.93) | | <0.001 |
| Diseases of the respiratory system, n (%) | 874 (1.1%) | 658 (1.6%) | 1.44 (1.30 - 1.59) | | <0.001 | 1.41 (1.27 - 1.56) | | <0.001 |
| **1-<18 years at inclusion** |  | | | | | | | |
| Sepsis, n (%) | 312 (2.2%) | 2 254 (16%) | 8.80 (7.81 - 9.94) | | <0.001 | 6.00 (5.30 - 6.80) | | <0.001 |
| Infectious diseases, n (%) | 961 (6.7%) | 2 128 (15%) | 2.54 (2.35 - 2.76) | | <0.001 | 1.79 (1.64 - 1.95) | | <0.001 |
| Neoplasms, n (%) | 107 (0.7%) | 223 (1.6%) | 2.19 (1.74 - 2.77) | | <0.001 | 1.05 (0.81 - 1.36) | | 0.719 |
| Mental and behavioural disorders, n (%) | 363 (2.5%) | 156 (1.1%) | 0.44 (0.36 - 0.53) | | <0.001 | 0.47 (0.39 - 0.57) | | <0.001 |
| Diseases of the circulatory system, n (%) | 501 (3.5%) | 388 (2.8%) | 0.80 (0.70 - 0.92) | | 0.001 | 0.68 (0.58 - 0.78) | | <0.001 |
| Diseases of the respiratory system, n (%) | 508 (3.5%) | 325 (2.3%) | 0.66 (0.57 - 0.76) | | <0.001 | 0.71 (0.62 - 0.82) | | <0.001 |
| **New diagnosis 3-12 months after discharge after index hospitalization** | | | | | | | | |
| **0-<1 years at inclusion** |  | | | | | | | |
| Sepsis, n (%) | 485 (0.6%) | 393 (1.0%) | 1.55 (1.35 - 1.77) | | <0.001 | 1.44 (1.26 - 1.65) | | <0.001 |
| Infectious diseases, n (%) | 12 653 (17%) | 6 712 (17%) | 1.01 (0.98 - 1.04) | | 0.521 | 1.00 (0.97 - 1.04) | | 0.784 |
| Neoplasms, n (%) | 553 (0.7%) | 264 (0.7%) | 0.91 (0.78 - 1.05) | | 0.195 | 0.85 (0.73 - 0.98) | | 0.03 |
| Mental and behavioural disorders, n (%) | 790 (1.0%) | 451 (1.1%) | 1.09 (0.97 - 1.22) | | 0.161 | 1.08 (0.96 - 1.21) | | 0.217 |
| Diseases of the circulatory system, n (%) | 2 411 (3.2%) | 1 173 (2.9%) | 0.92 (0.86 - 0.99) | | 0.027 | 0.91 (0.85 - 0.98) | | 0.013 |
| Diseases of the respiratory system, n (%) | 3 262 (4.3%) | 2 233 (5.6%) | 1.32 (1.25 - 1.39) | | <0.001 | 1.31 (1.24 - 1.39) | | <0.001 |
| **1-<18 years at inclusion** |  |  |  |  |  |  |  |  |
| Sepsis, n (%) | 266 (1.9%) | 684 (5.1%) | 2.77 (2.40 - 3.20) | | <0.001 | 1.67 (1.43 - 1.95) | | <0.001 |
| Infectious diseases, n (%) | 1 082 (7.7%) | 1 877 (14%) | 1.94 (1.79 - 2.10) | | <0.001 | 1.53 (1.41 - 1.67) | | <0.001 |
| Neoplasms, n (%) | 141 (1.0%) | 299 (2.2%) | 2.24 (1.83 - 2.75) | | <0.001 | 1.03 (0.83 - 1.30) | | 0.766 |
| Mental and behavioural disorders, n (%) | 593 (4.2%) | 305 (2.3%) | 0.53 (0.46 - 0.60) | | <0.001 | 0.55 (0.48 - 0.64) | | <0.001 |
| Diseases of the circulatory system, n (%) | 503 (3.6%) | 474 (3.5%) | 0.98 (0.86 - 1.12) | | 0.781 | 0.85 (0.74 - 0.98) | | 0.022 |
| Diseases of the respiratory system, n (%) | 592 (4.2%) | 519 (3.8%) | 0.91 (0.81 - 1.03) | | 0.128 | 0.91 (0.81 - 1.03) | | 0.155 |
| **New diagnosis 1-5 years after discharge after index hospitalization** | | | | | | | | |
| **0-<1 years at inclusion** |  | | | | | | | |
| Sepsis, n (%) | 610 (0.8%) | 407 (1.1%) | 1.27 (1.12 - 1.44) | | <0.001 | 1.23 (1.08 - 1.40) | | 0.001 |
| Infectious diseases, n (%) | 13 324 (18%) | 6 733 (17%) | 0.95 (0.92 - 0.98) | | 0.001 | 0.95 (0.92 - 0.98) | | 0.002 |
| Neoplasms, n (%) | 684 (0.9%) | 381 (1.0%) | 1.06 (0.93 - 1.20) | | 0.393 | 1.01 (0.89 - 1.15) | | 0.847 |
| Mental and behavioural disorders, n (%) | 2 979 (4.1%) | 1 721 (4.4%) | 1.10 (1.03 - 1.17) | | 0.002 | 1.09 (1.03 - 1.16) | | 0.005 |
| Diseases of the circulatory system, n (%) | 4 642 (6.3%) | 2 451 (6.3%) | 1.00 (0.95 - 1.05) | | 0.971 | 1.00 (0.95 - 1.05) | | 0.889 |
| Diseases of the respiratory system, n (%) | 10 488 (14%) | 5 724 (15%) | 1.04 (1.00 - 1.08) | | 0.025 | 1.04 (1.00 - 1.08) | | 0.027 |
| **1-<18 years at inclusion** |  | | | | | | | |
| Sepsis, n (%) | 281 (2.1%) | 432 (3.4%) | 1.64 (1.41 - 1.91) | | <0.001 | 1.39 (1.19 - 1.64) | | <0.001 |
| Infectious diseases, n (%) | 1 591 (12%) | 1 696 (13%) | 1.14 (1.06 - 1.23) | | <0.001 | 1.12 (1.04 - 1.21) | | 0.003 |
| Neoplasms, n (%) | 318 (2.4%) | 602 (4.7%) | 2.04 (1.78 - 2.34) | | <0.001 | 1.18 (1.02 - 1.38) | | 0.03 |
| Mental and behavioural disorders, n (%) | 1 442 (11%) | 1 050 (8.2%) | 0.74 (0.68 - 0.81) | | <0.001 | 0.76 (0.70 - 0.83) | | <0.001 |
| Diseases of the circulatory system, n (%) | 1 010 (7.5%) | 1 067 (8.4%) | 1.12 (1.02 - 1.23) | | 0.013 | 1.01 (0.92 - 1.12) | | 0.772 |
| Diseases of the respiratory system, n (%) | 984 (7.3%) | 1 137 (8.9%) | 1.24 (1.13 - 1.35) | | <0.001 | 1.20 (1.09 - 1.32) | | <0.001 |
| ^a^Adjusted comorbidities, age groups 1-<18 year also adjusted for age | | | | | | | | |

| eTable 9, Unemployment, Stepwise Logistic Regression Analysis | | | | | |  |  |  |
| --- | --- | --- | --- | --- | --- | --- | --- | --- |
|  | | | **Crude** | | | **Adjusted^a^** | | |
| **Group** | **Split** | **Variable** | **N** | **OR (95% CI)** | **P-value** | **N** | **OR (95% CI)** | **P-value** |
| **20 years of age** | 0 days, versus 1-365 days | Sepsis Cohort | 53 395 | 1.01 (0.97 - 1.05) | 0.736 | 53 316 | 0.99 (0.95 - 1.03) | 0.490 |
|  | 0-180 days, versus 181-365 days | Sepsis Cohort | 53 395 | 1.09 (0.94 - 1.26) | 0.273 | 53 316 | 1.03 (0.88 - 1.20) | 0.686 |
| **25 years of age** | 0 days, versus 1-365 days | Sepsis Cohort | 37 575 | 1.00 (0.94 - 1.07) | 0.943 | 37 516 | 0.97 (0.91 - 1.03) | 0.305 |
|  | 0-180 days, versus 181-365 days | Sepsis Cohort | 37 575 | 1.06 (0.90 - 1.26) | 0.479 | 37 516 | 1.03 (0.86 - 1.22) | 0.755 |
| **30 years of age** | 0 days, versus 1-365 days | Sepsis Cohort | 20 591 | 0.95 (0.87 - 1.05) | 0.316 | 20 562 | 0.94 (0.86 - 1.03) | 0.192 |
|  | 0-180 days, versus 181-365 days | Sepsis Cohort | 20 591 | 0.90 (0.75 - 1.08) | 0.264 | 20 562 | 0.89 (0.74 - 1.07) | 0.206 |
| ^a^Adjusted for age, sex and comorbidities | | | | | | | | |

## eTable 10, Occurrence of Disability Pension, Logistic Regression Analysis

|  | | **Crude** | | | **Adjusted^a^** | | |
| --- | --- | --- | --- | --- | --- | --- | --- |
| **Group** | **Variable** | **N** | **OR (95% CI)** | **P-value** | **N** | **OR (95% CI)** | **P-value** |
| 20 år | Sepsis Cohort | 53 559 | 0.89 (0.84 - 0.94) | <0.001 | 53 480 | 0.85 (0.80 - 0.90) | <0.001 |
| 25 år | Sepsis Cohort | 37 575 | 0.82 (0.77 - 0.88) | <0.001 | 37 516 | 0.80 (0.75 - 0.86) | <0.001 |
| 30 år | Sepsis Cohort | 20 591 | 0.78 (0.71 - 0.85) | <0.001 | 20 562 | 0.75 (0.68 - 0.82) | <0.001 |
| ^a^Adjusted for age, sex and comorbidities | | | | | | | |
